# Correction: Human and Murine Clonal CD8+ T Cell Expansions Arise during Tuberculosis Because of TCR Selection

**DOI:** 10.1371/journal.ppat.1005144

**Published:** 2015-09-17

**Authors:** The PLOS Pathogens Staff

## Notice of Republication

This article was republished on August 12, 2015, to correct typographical errors throughout the text, in the Abstract, and in Table 2 that were introduced during the typesetting process. The errors consisted of un-subscripted characters and omitted Greek symbols. The publisher apologizes for the errors. Please download this article again to view the correct version. The originally published, uncorrected article and the republished, corrected article are provided here for reference.

## Supporting Information

S1 File Originally published, uncorrected article. (PDF)

S2 File Republished, corrected article. (PDF)
