# Supplementary material for: Human and Murine Clonal CD8+ T Cell Expansions Arise during Tuberculosis Because of TCR Selection
Source: PLoS Pathog. 2015 May 6;11(5):e1004849. doi: 10.1371/journal.ppat.1004849 (PMC4422591; doi:10.1371/journal.ppat.1004849)
Supplement: S1 Data — Summary of deep sequencing data of human TCRβs from lung granulomas and lymph node resected from subjects with tuberculosis. (PDF) [file ppat.1004849.s001.pdf]

Supplemental Data 1: Deep Sequencing of human TCRs from TB granulomas

Summary of deep sequencing data of human TCRβs from lung granulomas and lymph node resected from subjects with tuberculosis.

Sample Name	Total	Unique	Productive Total	Productive Uniques	Entropy	Clonality	Max Frequency
**21LGCD8A**	23,756	392	15,799	265	7.38	0.08	3.23
**23LGCD8A**	91,102	1,366	76,127	1,051	8.06	0.20	7.66
**23LGCD8B**	273,696	3,927	230,446	3,098	9.23	0.20	7.05
**23LGCD8C**	161,402	2,146	136,091	1,695	8.52	0.21	5.07
**23LNCD81**	894,931	28,840	741,259	23,425	12.70	0.13	2.88
**24LGCD8A**	140,292	3,834	116,101	3,061	9.95	0.14	2.04
**24LGCD8B**	239,076	4,734	197,682	3,759	9.63	0.19	2.79
**24LGCD8C**	108,174	1,649	85,231	1,291	9.14	0.12	2.49
**26LGCD8A**	1,052,806	15,597	868,384	12,513	11.09	0.19	2.27
**26LGCD8C**	487,494	10,276	416,991	8,313	9.96	0.23	4.24
**27LGCD8A**	14,979	415	9,661	279	7.25	0.11	3.77
**27LGCD8B**	411,965	3,953	313,033	2,994	9.60	0.17	3.11

Total: number of individual reads
Unique: Number of unique sequences
Productive: Number of total sequences that correspond to a productive TCRB (no missense or nonsense substitutions)
Productive unique: number of unique productive sequences
Entropy: a measure of diversity of the data set (see methods)
Clonality: a measure of the similarity of sequences to each other (see methods)
Max frequency: the highest frequency of a sequence detected
