# Supplementary material for: Human and Murine Clonal CD8+ T Cell Expansions Arise during Tuberculosis Because of TCR Selection
Source: PLoS Pathog. 2015 May 6;11(5):e1004849. doi: 10.1371/journal.ppat.1004849 (PMC4422591; doi:10.1371/journal.ppat.1004849)
Supplement: S2 Data — A. The Vβ gene distribution is shown for two samples: (top) CD8+ T cells from a human lung granulomas; and (B) PBMC from a normal donor. One can see how differences in the Vβ gene use affects the calculation of clonality can be visualized. Numbers do not add to 100% because only productive recombination events are plotted. Width axis: V gene; Depth Axis: CDR3 length; Height axis: frequency. B. The Vβ gene distribution is shown for four samples from a single subject (#23): lung sample A, B, and C; and LN. Although there are considerable differences between the samples, particularly with respect to TCR diversity (see Fig 1), the most abundant TCRs are shared between the different lung lesions and to a lesser extent, the LN sample. Again, one can visualize how the Vβ gene use affects clonality. Width axis: V gene; Depth Axis: CDR3 length; Height axis: frequency. (PDF) [file ppat.1004849.s002.pdf]

Supplemental Data 2: Clonality and Clonal Distribution

A.

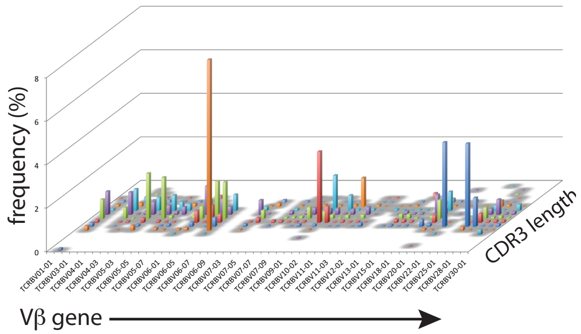

#23 LUNG granuloma A
clonality = 0.20
maximum frequency = 7.66%

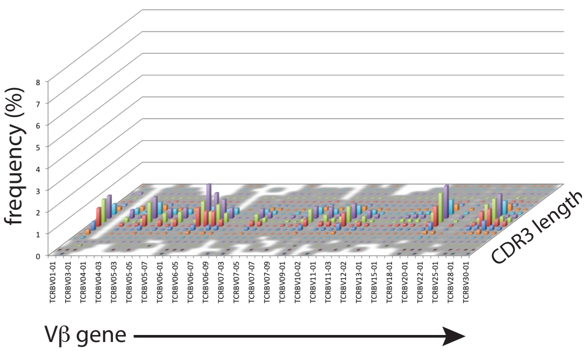

HPI13939 PBMC
clonality = 0.07
maximum frequency = 0.29%

B.

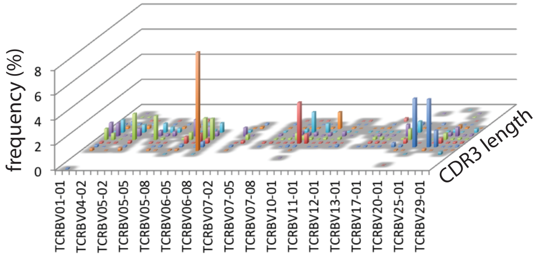

#23 LUNG granuloma A
clonality = 0.20
maximum frequency = 7.66%

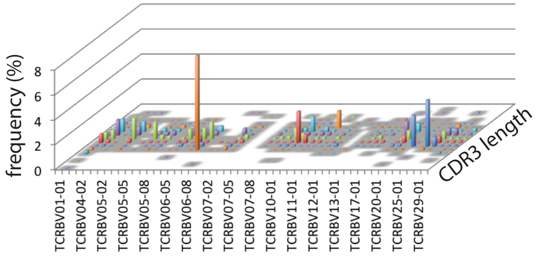

#23 LUNG granuloma B
clonality = 0.20
maximum frequency = 7.05%

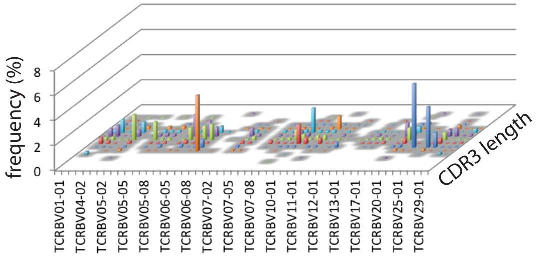

#23 LUNG granuloma C
clonality = 0.21
maximum frequency = 5.07%

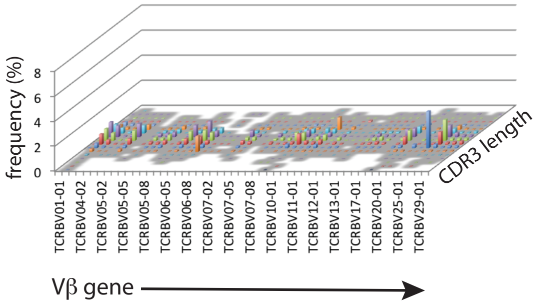

#23 lymph node
clonality = 0.13
maximum frequency = 2.88%

A. The Vβ gene distribution is shown for two samples: (top) CD8+ T cells from a human lung granulomas; and (B) PBMC from a normal donor. One can see how differences in the Vβ gene use affects the calculation of clonality can be visualized. Numbers do not add to 100% because of only productive recombination events are plotted. Width axis: V gene; Depth Axis: CDR3 length; Height axis: frequency.

B. The Vβ gene distribution is shown for four samples from a single subject (#23): lung sample A, B, and C; and LN. Although there are considerable differences between the samples, particularly with respect to TCR diversity (see Figure 1), the most abundant TCRs are shared between the different lung lesions and to a lesser extent, the LN sample. Again, one can visualize how the Vβ gene use affects clonality. Width axis: V gene; Depth Axis: CDR3 length; Height axis: frequency.
