# Supplementary material for: Human and Murine Clonal CD8+ T Cell Expansions Arise during Tuberculosis Because of TCR Selection
Source: PLoS Pathog. 2015 May 6;11(5):e1004849. doi: 10.1371/journal.ppat.1004849 (PMC4422591; doi:10.1371/journal.ppat.1004849)
Supplement: S3 Data — The TCRβ frequencies, derived for the frequency of unique DNA sequences, in each sample analyzed by deep sequencing. The bar is the median and the error bars denote the interquartile range. For comparison, the TCRβ frequencies in PBMC from three normal donors are shown. Only productive recombination events are plotted. (PDF) [file ppat.1004849.s003.pdf]

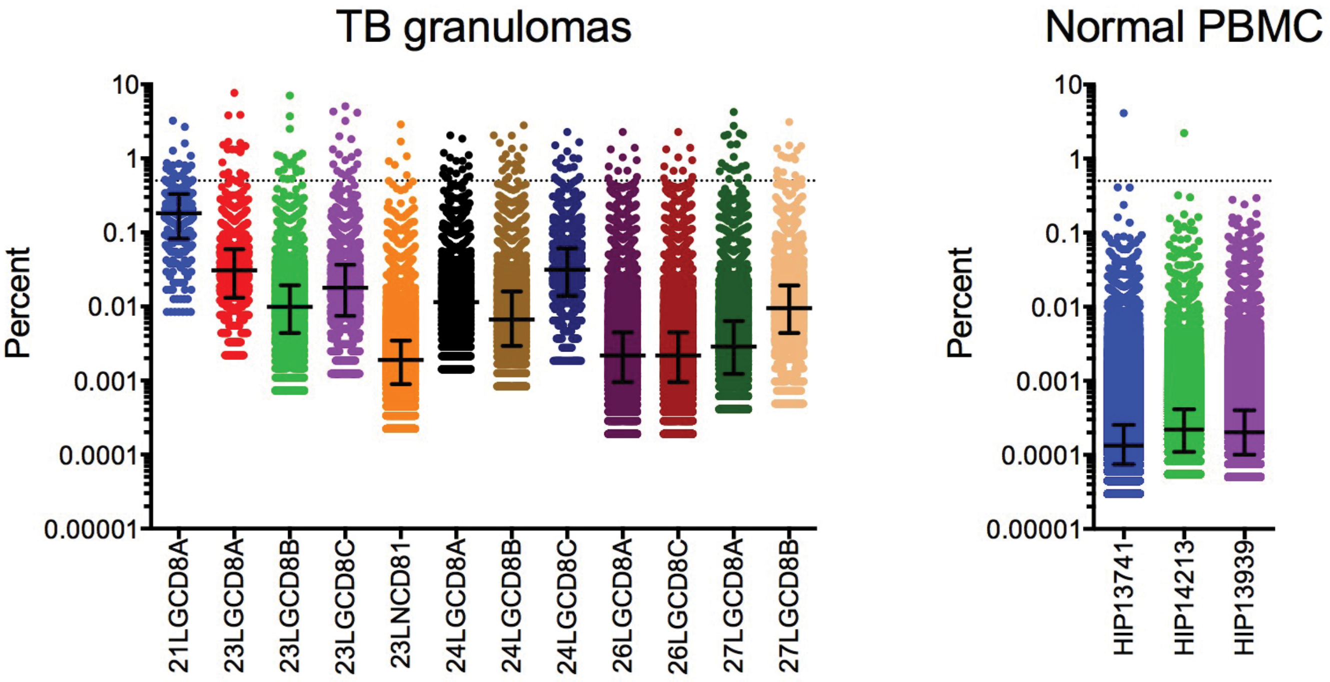

Supplemental Data 3: TCRβ frequencies in human lung granulomas

The TCRβ frequencies, derived for the frequency of unique DNA sequences, in each sample analyzed by deep sequencing. The bar is the median and the error bars denote the interquartile range. For comparison, the TCRβ frequencies in PBMC from three normal donors are shown. Only productive recombination events are plotted.
