# Supplementary material for: Human and Murine Clonal CD8+ T Cell Expansions Arise during Tuberculosis Because of TCR Selection
Source: PLoS Pathog. 2015 May 6;11(5):e1004849. doi: 10.1371/journal.ppat.1004849 (PMC4422591; doi:10.1371/journal.ppat.1004849)
Supplement: S4 Data — The frequency of the “DREN” motif in the CDR3 region of TCRβ chains sequenced from human granulomas or PBMC samples from normal individuals. Multiple unique sequences were detected in these samples. The frequency of “DREN” motifs was elevated in lung granuloma compared to peripheral blood. Statistical testing after log10 transformation showed that these differences were significant (P<0.001) for all pairwise combinations by one-way ANOVA with Tukey’s multiple comparison test. These data show that TCRβ chains with the “DREN” CDR3 motif are increased in frequency among CD8+ T cells in tuberculous lung granulomas compared to peripheral blood T cells from healthy controls. (PDF) [file ppat.1004849.s004.pdf]

Supplemental Data 4: DREN motifs in human TCRs

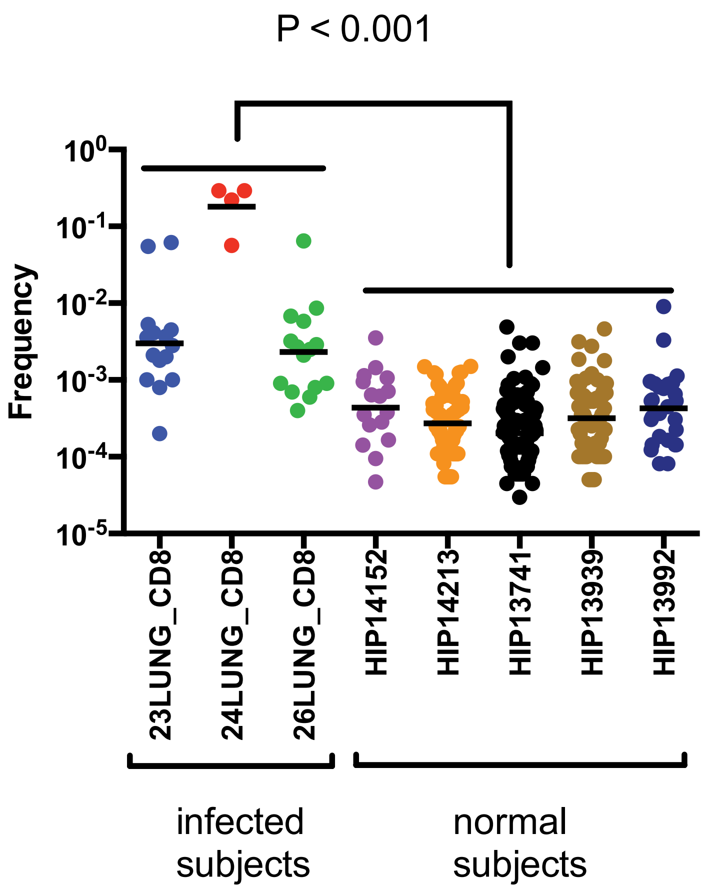

The frequency of the "DREN" motif in the CDR3 region of TCRB chains sequenced from human granulomas or PBMC samples from normal individuals. Multiple unique sequences were detected in these samples. The frequency of "DREN" motifs was elevated in lung granuloma compared to peripheral blood. Statistical testing after log10 transformation showed that these differences were significant ($P<0.001$) for all pairwise combinations by one-way ANOVA with Tukey's multiple comparison test. These data show that TCRβ chains with the "DREN" CDR3 motif are increased in frequency among CD8+ T cells in tuberculous lung granulomas compared to peripheral blood T cells from healthy controls.
