# Supplementary material for: Human and Murine Clonal CD8+ T Cell Expansions Arise during Tuberculosis Because of TCR Selection
Source: PLoS Pathog. 2015 May 6;11(5):e1004849. doi: 10.1371/journal.ppat.1004849 (PMC4422591; doi:10.1371/journal.ppat.1004849)
Supplement: S5 Data — A. Schematic of the Vα2var locus of Vα2var mice. B. The TB10.44-11-specific CD8+ T cell response following aerosol infection of WT vs. Vα2var mice. C. Survival of C57BL/6, Vα2var, or TCRα knockout mice after low dose aerosolized Mtb. (PDF) [file ppat.1004849.s005.pdf]

Supplemental Data 5: Infection of Vα2var mice with Mtb

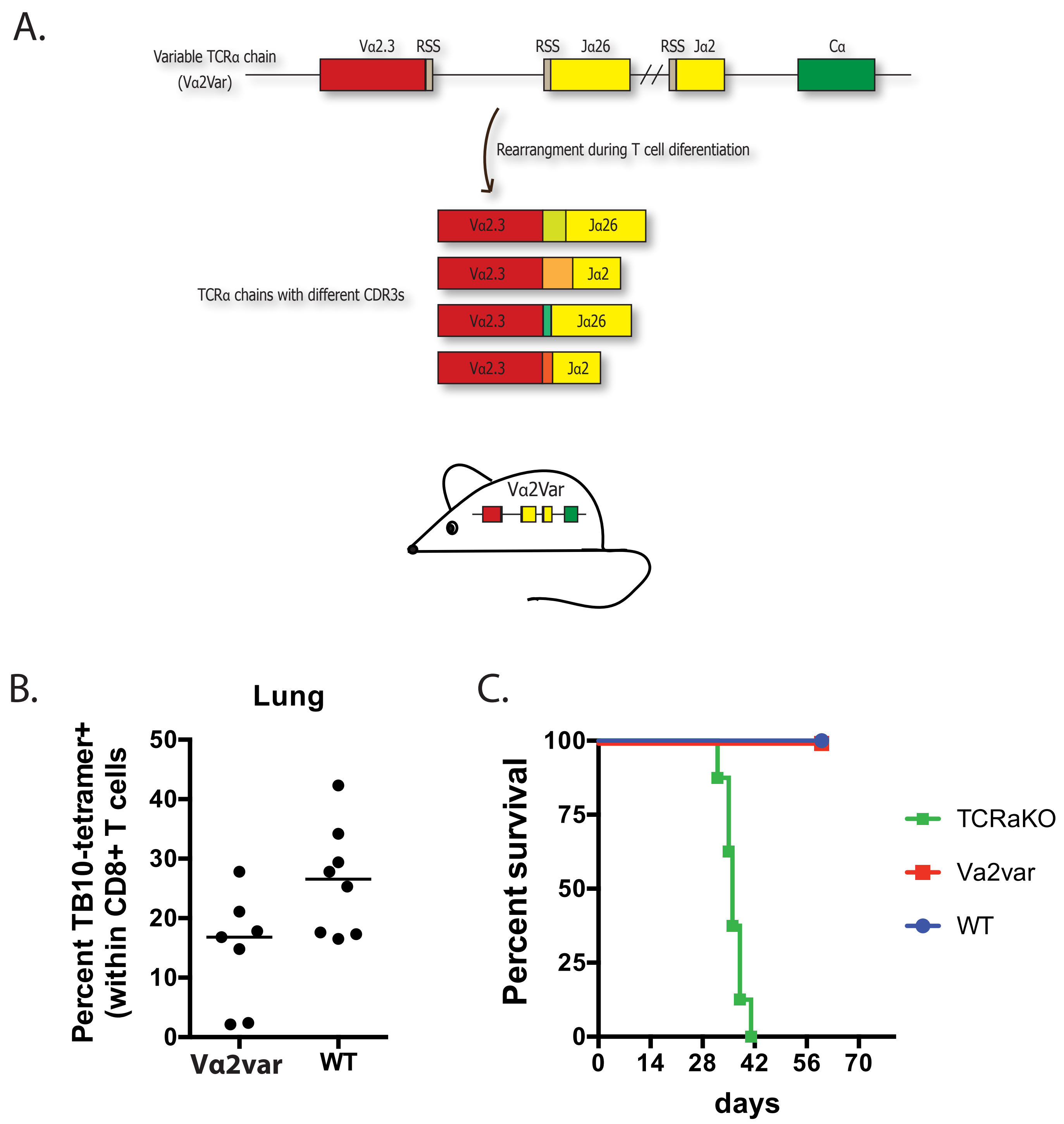

A. Schematic of the Vα2var locus of Vα2var mice.
B. The TB10.$4_{4\text{-}11}$-specific CD8+ T cell response following Mtb infection of WT vs. Vα2var mice.
C. Survival of C57BL/6, Vα2var, or TCRα knockout mice after low dose aerosolized Mtb.
