# Supplementary material for: Human and Murine Clonal CD8+ T Cell Expansions Arise during Tuberculosis Because of TCR Selection
Source: PLoS Pathog. 2015 May 6;11(5):e1004849. doi: 10.1371/journal.ppat.1004849 (PMC4422591; doi:10.1371/journal.ppat.1004849)
Supplement: S6 Data — To determine whether the enrichment of “R” or “D” was significant, we compared their occurrence in the normal TCRβ repertoire. We queried the splenic TCRβ repertoire from three C57BL/6 mice representing over 1.1 million reads and ~53,000 unique sequences each. The average frequency of “R”, “D”, or “RD” at CDR3β position 6, 7, or 6–7, was 10.1%, 7.5%, and 2.5% respectively, indicating “R,” “D,” and “RD,” were significantly enriched in the clonally expanded TB10.44-11-specific CD8+ T cells. Testing using the Chi-square with Yates' correction shows that the frequency of R, D, or RD in the clonally expanded sequences is significantly increased in the four cloned sequences compared to the normal splenic repertoire of B6 mice (P<0.0001). (PDF) [file ppat.1004849.s006.pdf]

Supplemental Data 6: Frequency of arginine and aspartic acid in naïve cells

Results are averaged from the TCR repertoire from 3 B6 spleens			
**Number of unique sequences**	53,499	53,499	53,499
**Query**	"R" @ 6	"D" @ 7	"RD" @ 6-7
**Number of positive results**	5,381	4,025	1,320
**Percentage of unique sequences with "positive" result**	10.1%	7.5%	2.5%

To determine whether the enrichment of "R" or "D" was significant, we compared their occurrence in the normal TCRβ repertoire. We queried the splenic TCRβ repertoire from three C57BL/6 mice representing over 1.1 million reads and ~53,000 unique sequences each. The average frequency of "R", "D", or "RD" at CDR3β position 6, 7, or 6-7, was 10.1%, 7.5%, and 2.5% respectively, indicating "R," "D," and "RD," were significantly enriched in the clonally expanded $TB10.4_{4\text{-}11}$-specific $CD8^+$ T cells. Testing using the Chi-square with Yates' correction shows that the frequency of R, D, or RD in the clonally expanded sequences is significantly increased in the four cloned sequences compared to the normal splenic repertoire of B6 mice ($P<0.0001$).
