# Supplementary material for: Human and Murine Clonal CD8+ T Cell Expansions Arise during Tuberculosis Because of TCR Selection
Source: PLoS Pathog. 2015 May 6;11(5):e1004849. doi: 10.1371/journal.ppat.1004849 (PMC4422591; doi:10.1371/journal.ppat.1004849)
Supplement: S7 Data — Rg3 CD8+ T cells were transferred to Mtb infected mice as described in the methods. Three to six weeks after aerosolized Mtb infection, lung cells were stimulated with the TB10.4 epitope. Intracellular staining for IFNγ, IL-2, TNF, and granzyme were performed. The results shown are gate on Rg3 CD8+ T cells identified by their co-expression of GFP and Vα2 (PDF) [file ppat.1004849.s007.pdf]

Supplemental Data 7: Rg3 CD8⁺ T cells express cytokines and granzyme B.

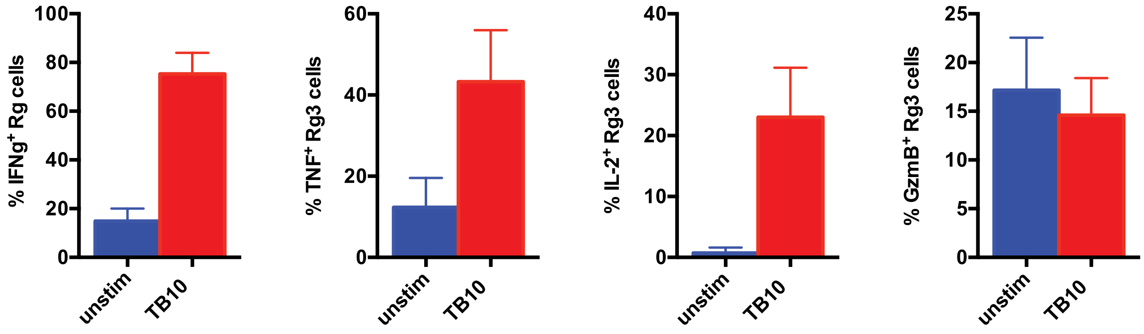

Rg3 CD8+ T cells were transferred to Mtb infected mice as described in the methods. Three to six weeks after aerosolized Mtb infection, lung cells were stimulated with the TB10.4 epitope. Intracellular staining for IFNg, IL-2, TNF, and granzyme were performed. The results shown are gate on Rg3 CD8+ T cells identified by their co-expression of GFP and Va2.
