# Supplementary material for: Human and Murine Clonal CD8+ T Cell Expansions Arise during Tuberculosis Because of TCR Selection
Source: PLoS Pathog. 2015 May 6;11(5):e1004849. doi: 10.1371/journal.ppat.1004849 (PMC4422591; doi:10.1371/journal.ppat.1004849)
Supplement: S8 Data — A. Protection mediated by 106, 105 or 104 in vitro activated Rg4 (e.g., TCR4) CD8+ T cells. T cells were transferred by the IV route into sublethally irradiated mice and challenged with Mtb. CFU were determined ~ 4 wks after infection. Protection (Δlog10) is the lung CFU in mice that did not receive Rg T cells minus the lung CFU in mice that did receive Rg T cells. Two independent experiments are shown each with 5 mice. One way ANOVA with Tukey’s post test to compare differences in CFU. *, p<0.05; ***, p<0.001. B. The level of TCRα staining on Rg3 vs. Rg4 CD8+ T cells from the lungs of infected mice. T test showed the difference was not significant. C. Priming of naïve Rg4 and Rg3 CD8+ T cells in the pulmonary LN of Mtb infected mice occurs with similar kinetics. Rg3 and Rg4 CD8+ T cells were co-transferred or Rg3 CD8+ T cells were transferred alone. Priming is indicated by acquisition of an activated (CD44+CD62Llo phenotype. (PDF) [file ppat.1004849.s008.pdf]

Supplemental Data 8: Rg4 CD8+ T cells mediate protection and are primed in the lungs of Mtb infected mice

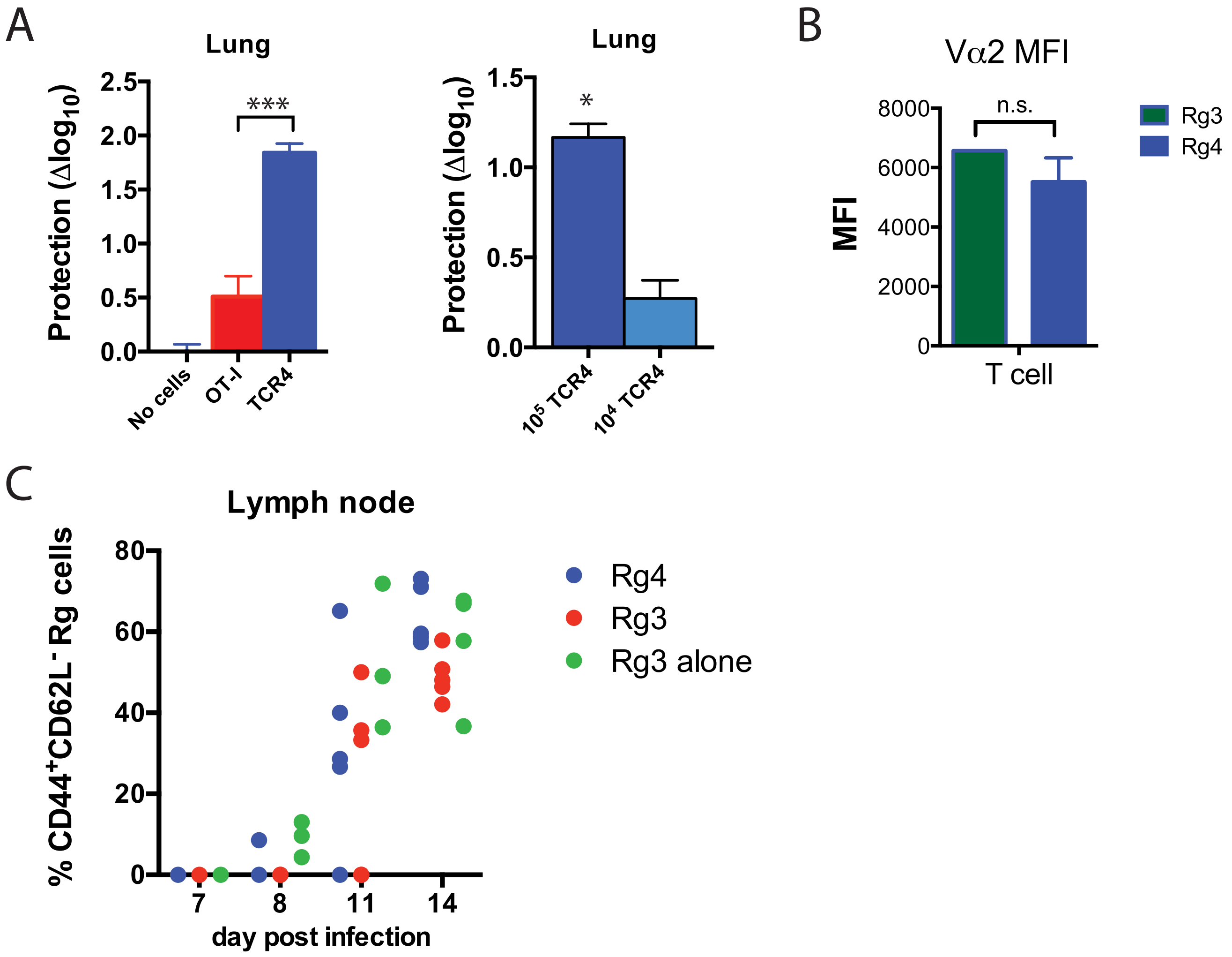

A. Protection mediated by 10^6, 10^5 or 10^4 in vitro activated Rg4 (e.g., TCR4) $CD8^+$ T cells. T cells were transferred by the IV route into sublethally irradiated mice and challenged with Mtb. CFU were determined ~ 4 wks after infection. Protection (Δlog10) is the lung CFU in mice that did not receive Rg T cells minus the lung CFU in mice that did receive Rg T cells. Two independent experiments are shown each with 5 mice. One way ANOVA with Tukey's post test to compare differences in CFU. *, $p<0.05$; ***, $p<0.001$.

B. The level of TCRα staining on Rg3 vs. Rg4 CD8+ T cells from the lungs of infected mice. T test showed the difference was not significant.

C. Priming of naïve Rg4 and Rg3 CD8+ T cells in the pulmonary LN of Mtb infected mice occurs with similar kinetics. Rg3 and Rg4 CD8+ T cells were co-transferred or Rg3 CD8+ T cells were transferred alone. Priming is indicated by acquisition of an activated ($CD44^+CD62L^{lo}$ phenotype.
