# Supplementary material for: Human and Murine Clonal CD8+ T Cell Expansions Arise during Tuberculosis Because of TCR Selection
Source: PLoS Pathog. 2015 May 6;11(5):e1004849. doi: 10.1371/journal.ppat.1004849 (PMC4422591; doi:10.1371/journal.ppat.1004849)
Supplement: S9 Data — List of the TCRα and TCRβ primers used for nested PCR for the single cell analysis of TCRs used by TB10-specific CD8+ T cells from the lungs of Mtb-infected mice. (PDF) [file ppat.1004849.s009.pdf]

Supplemental Data 9: Primers used for single cell PCR analysis of TCRs used by murine CD8+ T cells

Name	Sequence	Name	Sequence
TRAV1_int	TGGATGGTTTGAAGGACAGTGGGC	TRAV1_ext	agcaacgtgaaggccaagcc
TRAV2_int	caaggatataaggactatgtgg	TRAV2_ext	caagaagtgaacgtgtcctg
TRAV3_int	cgaaggacaaggattcactg	TRAV3_ext	TATCATYTGCACCTACACAGAC
TRAV4_int	AACAAAGGAGAATGGRAGG	TRAV4_ext	CTGCTCTGAGATGCAATTTTWC
TRAV5_int	gcagatccaaggactcatcg	TRAV5_ext	TGGTATAAGCAAGAACCTGG
TRAV6_int	ctcagtgcatgagtcagactc	TRAV6_ext	TATCCCGGAGAAGGTCCACAGCTC
TRAV6D-6_int	TGTTCGATAYCYTGGAGAAGG	TRAV6D-6_ext	GCACGTATTCAGCCACAAGCATAGG
TRAV7_int	TGGTACAGACAGCATYCTGG	TRAV7_ext	agcagagcccagaatccctc
TRAV8_int	GTTCAAATGAGMGAGAGAAG	TRAV8_ext	cagtggtacagacagaagtcag
TRAV9_int	TGCTCCTCAAGTACTATTCVGG	TRAV9_ext	AGCTGAGATGCAASTATTCCT
TRAV10_int	GCTGGAAAGGGTCTCCACTTTGTG	TRAV10_ext	GTGTTCGAGAGGGAGACAGCGC
TRAV11_int	gtcaaatgggagatactcagc	TRAV11_ext	ggtggttcaaacaggacac
TRAV12_int	gccactctccataagagcag	TRAV12_ext	ctgtgatgctgaactgcacc
TRAV13_int	CTTTGMMYATWTCCTCCTCC	TRAV13_ext	CAGTGGTTTTACCAAMRTCCT
TRAV14_int	cacaatcttcttcaataaaaggg	TRAV14_ext	cccaatctctgacagtctgg
TRAV15_int	gccgctattctgtagtcttc	TRAV15_ext	gtgattcaggtctggtcaac
TRAV16_int	CAGCAAGTGGGRAAATAGTTTT	TRAV16_ext	gaagacaacggtgacaatgg
TRAV17_int	CCAGAGCCTCCAGTTTCTCC	TRAV17_ext	catacagtgcagcaccttacc
TRAV19_int	cacactcctgatatccgtac	TRAV19_ext	gatatgtttgactattttgcctg
TRAV20_int	agaagggaagattcgaggtg	TRAV20_ext	tcacgctcctaatagacattc
TRAV21_int	GTGACTCACGGTCTACAACAAAA	TRAV21_ext	ccagattcaatggaaagtactg
VB1_Int	ctttggaatgtgagcaacatc	VB1_Ext	ggaaacagcactcatgaacac
VB2_Int	cttgaagaattcccagtatccc	VB2_Ext	ctacagaccccacagtgac
VB3_Int	cttcagcaaatagacatgactg	VB3_Ext	caagatatctggtgaaagggc
VB4_Int	cttatggacaatcagactgcc	VB4_Ext	agtatctaggccacaatgc
VB5_Int	gcagattctcagtccaacag	VB5_Ext	ctcytgggaacaagttcagc
VB6_Int	cagggaaaggattgagactg	VB6_Ext	cacatggtgatggtggcatc
VB7_Int	gaatgtggacaggacatgag	VB7_Ext	gaacaggccttgtggacatg
VB8_Int	atgtactggtatcggcaggac	VB8_Ext	tgkgwrcaaaacacatggaggc
VB9_Int	gcaagagttggaaaaccagtg	VB9_Ext	tgcagccacttttgtggatac
VB10_Int	gtaaacgaaacagttccaaggc	VB10_Ext	aattgctgaagattatgtttagc
VB11_Int	caagaagcaactctgtggtg	VB11_Ext	gagagcagaaccaacaaatgc
VB12_Int	ctaaattcatccttctccactc	VB12_Ext	gcaagtctcttatggaagatgg
VB13_Int	ctgtttccttttggtgtgacc	VB13_Ext	gttccttgacacagtactgtc
VB14_Int	ctaacctctactggtactgg	VB14_Ext	gctcagactatccatcaatgg
VB15_Int	gaagaaccatctgtaagagtgg	VB15_Ext	ttctggggcctggctgtg
VB16_Int	gtgacccagtttctaatcacc	VB16_Ext	gaagcaggacacacaggac
VB17_Int	cctggtcaaagagaaaggac	VB17_Ext	gcagctctttatgttgctgg
VB18_Int	gctgacagtcaagttgtttcg	VB18_Ext	tgtcctcctctaccaaaagc
VB19_Int	ctacaagaaaccgggagaag	VB19_Ext	ctctggggttgtccagaatc
CaR-Int	CGGCACATTGATTTGGGAGTC	CaR_Ext	GGCCCCATTGCTCTTGGAATC
CBR-Int	aagcccctggccaagcacac	CBR_Ext	ctataattgctctccttgtagg

List of the TCRα and TCRβ primers used for nested PCR for the single cell analysis of TCRs used by TB10-specific CD8+ T cells from the lungs of Mtb-infected mice.
