# Supplementary material for: Human and Murine Clonal CD8+ T Cell Expansions Arise during Tuberculosis Because of TCR Selection
Source: PLoS Pathog. 2015 May 6;11(5):e1004849. doi: 10.1371/journal.ppat.1004849 (PMC4422591; doi:10.1371/journal.ppat.1004849)
Supplement: S10 Data — TCR sequences and 2A peptide cloned into retroviral vectors. Sequences of four TCR sequences as assembled and cloned into the retroviral vectors. The first sequence is color-coded. VαJα (blue); Cα (purple); P2A (grey); VβDJβ (yellow); Cβ (green). (PDF) [file ppat.1004849.s010.pdf]

Supplemental Data 10: Retrogenic TCR sequences. TCR sequences and 2A peptide cloned into retroviral vectors.

Sequences of four TCR sequences as assembled and cloned into the retroviral vectors. The first sequence is color-coded. VαJα (blue); Cα (purple); P2A (grey); VβDJβ (yellow); Cβ (green)

TCR1

ATGGACAAGATCCTGACAGCAACGTTTTTACTCCTAGGCCTTCACCTAGCTGGGGTGAATGGCCAGCAGCAGGAGAA

ACGTGACCAGCAGCAGGTGAGACAAAGTCCCCAATCTCTGACAGTCTGGGAAGGAGAGACCGCAATTCTGAACTGCA

GTTATGAGGACAGCACTTTTAACTACTTCCCATGGTACCAGCAGTTCCCTGGGGAAGGCCCTGCACTCCTGATATCC

ATACGTTCAGTGTCCGATAAAAAGGAAGATGGACGATTCACAATCTTCTTCAATAAAAGGGAGAAAAAGCTCTCCTT

GCACATCACAGACTCTCAGCCTGGAGACTCAGCTACCTACTTCTGTGCAGATACTGGAGGACTAAGTGGTAAATTAA

CATTCGGGGAAGGGACCCAAGTGACGGTAATATCTGACATCCAGAACCCAGAACCTGCTGTGTACCAGTTAAAAGAT

CCTCGGTCTCAGGACAGCACCCTCTGCCTGTTCACCGACTTTGACTCCCAAATCAATGTGCCGAAAACCATGGAATC

TGGAACGTTCATCACTGACAAAACTGTGCTGGACATGAAAGCTATGGATTCCAAGAGCAATGGGGCCATTGCCTGGA

GCAACCAGACAAGCTTCACCTGCCAAGATATCTTCAAAGAGACCAACGCCACCTACCCCAGTTCAGACGTTCCCTGT

GATGCCACGTTGACCGAGAAAAGCTTTGAAACAGATATGAACCTAAACTTTCAAAACCTGTCAGTTATGGGACTCCG

AATCCTCCTGCTGAAAGTAGCGGGATTTAACCTGCTCATGACGCTGAGGCTGTGGTCCGGCTCCGGAGCCACGAACT

TCTCTCTGTTAAAGCAAGCAGGAGACGTGGAAGAAAACCCCGGTCCCATGTTACTGCTTCTATTACTTCTGGGGCCT

GGCTGTGGGCTTGGAGCACTCGTCTATCAATATCCCAGAAGAACCATCTGTAAGAGTGGAACTTCCATGAGGATGGA

GTGTCAAGCTGTGGGTTTTCAGGCAACTTCTGTAGCTTGGTATCGTCAATCGCCTCAAAAGACATTTGAACTGATAG

CACTTTCTACTGTGAACTCAGCAATCAAATATGAACAAAATTTTACCCAGGAAAAATTTCCCATCAGTCATCCCAAC

TTATCCTTTTCATCTATGACAGTTTTAAATGCATATCTTGAAGACAGAGGCTTATATCTCTGTGGTGCTAGGGATCG

ACGGACAGGGGGAGAAACGCTGTATTTTGGCTCAGGAACCAGACTGACTGTTCTCGAGGATCTGAGAAATGTGACTC

CACCCAAGGTCTCCTTGTTTGAGCCATCAAAAGCAGAGATTGCAAACAAACAAAAGGCTACCCTCGTGTGCTTGGCC

AGGGGCTTCTTCCCTGACCACGTGGAGCTGAGCTGGTGGGTGAATGGCAAGGAGGTCCACAGTGGGGTCAGCACGGA

CCCTCAGGCCTACAAGGAGAGCAATTATAGCTACTGCCTGAGCAGCCGCCTGAGGGTCTCTGCTACCTTCTGGCACA

ATCCTCGCAACCACTTCCGCTGCCAAGTGCAGTTCCATGGGCTTTCAGAGGAGGACAAGTGGCCAGAGGGCTCACCC

AAACCTGTCACACAGAACATCAGTGCAGAGGCCTGGGGCCGAGCAGACTGTGGGATTACCTCAGCATCCTATCAACA

AGGGGTCTTGTCTGCCACCATCCTCTATGAGATCCTGCTAGGGAAAGCCACCCTGTATGCTGTGCTTGTCAGTACAC

TGGTGGTGATGGCTATGGTCAAAAGAAAGAATTCATGA

VaJa/Ca P2A VbDJb/Cb

TCR2

ATGGACAAGATCCTGACAGCAACGTTTTTACTCCTAGGCCTTCACCTAGCTGGGGTGAATGGCCAGCAGCAGGAGAA

ACGTGACCAGCAGCAGGTGAGACAAAGTCCCCAATCTCTGACAGTCTGGGAAGGAGAGACCGCAATTCTGAACTGCA

GTTATGAGGACAGCACTTTTAACTACTTCCCATGGTACCAGCAGTTCCCTGGGGAAGGCCCTGCACTCCTGATATCC

ATACGTTCAGTGTCCGATAAAAAGGAAGATGGACGATTCACAATCTTCTTCAATAAAAGGGAGAAAAAGCTCTCCTT

GCACATCACAGACTCTCAGCCTGGAGACTCAGCTACCTACTTCTGTGCAGCAAGTCGGGGAACTGGAGGACTAAGTG

GTAAATTAACATTCGGGGAAGGGACCCAAGTGACGGTAATATCTGACATCCAGAACCCAGAACCTGCTGTGTACCAG

TTAAAAGATCCTCGGTCTCAGGACAGCACCCTCTGCCTGTTCACCGACTTTGACTCCCAAATCAATGTGCCGAAAAC

CATGGAATCTGGAACGTTCATCACTGACAAAACTGTGCTGGACATGAAAGCTATGGATTCCAAGAGCAATGGGGCCA

TTGCCTGGAGCAACCAGACAAGCTTCACCTGCCAAGATATCTTCAAAGAGACCAACGCCACCTACCCCAGTTCAGAC

GTTCCCTGTGATGCCACGTTGACCGAGAAAAGCTTTGAAACAGATATGAACCTAAACTTTCAAAACCTGTCAGTTAT

GGGACTCCGAATCCTCCTGCTGAAAGTAGCGGGATTTAACCTGCTCATGACGCTGAGGCTGTGGTCCGGCTCCGGAG

CCACGAACTTCTCTCTGTTAAAGCAAGCAGGAGACGTGGAAGAAAACCCCGGTCCCATGGATATCTGGCTTCTAGGT

TGGATAATTTTTAGTTTCTTGGAAGCAGGACACACAGGACCCAAAGTCTTACAGATCCCAAGTCATCAAATAATAGA

TATGGGGCAGATGGTGACCCTCAATTGTGACCCAGTTTCTAATCACCTATATTTTTATTGGTATAAACAGATTTTAG

GACAGCAGATGGAGTTTCTGGTTAATTTCTACAATGGTAAAGTCATGGAGAAGTCTAAACTGTTTAAGGATCAGTTT

TCAGTTGAAAGACCAGATGGTTCATATTTCACTCTGAAAATCCAACCCACAGCACTGGAGGACTCAGCTGTGTACTT

CTGTGCCAGCAGCGCTCGGGACTGGGGCGACACCCAGTACTTTGGGCCAGGCACTCGGCTCCTCGTGTTAGAGGATC

TGAGAAATGTGACTCCACCCAAGGTCTCCTTGTTTGAGCCATCAAAAGCAGAGATTGCAAACAAACAAAAGGCTACC

CTCGTGTGCTTGGCCAGGGGCTTCTTCCCTGACCACGTGGAGCTGAGCTGGTGGGTGAATGGCAAGGAGGTCCACAG

TGGGGTCAGCACGGACCCTCAGGCCTACAAGGAGAGCAATTATAGCTACTGCCTGAGCAGCCGCCTGAGGGTCTCTG

CTACCTTCTGGCACAATCCTCGCAACCACTTCCGCTGCCAAGTGCAGTTCCATGGGCTTTCAGAGGAGGACAAGTGG

CCAGAGGGCTCACCCAAACCTGTCACACAGAACATCAGTGCAGAGGCCTGGGGCCGAGCAGACTGTGGGATTACCTC

AGCATCCTATCAACAAGGGGTCTTGTCTGCCACCATCCTCTATGAGATCCTGCTAGGGAAAGCCACCCTGTATGCTG

TGCTTGTCAGTACACTGGTGGTGATGGCTATGGTCAAAAGAAAGAATTCATGA

TCR3

ATGGACAAGATCCTGACAGCAACGTTTTTACTCCTAGGCCTTCACCTAGCTGGGGTGAATGGCCAGCAGCAGGAGAA

ACGTGACCAGCAGCAGGTGAGACAAAGTCCCCAATCTCTGACAGTCTGGGAAGGAGAGACCGCAATTCTGAACTGCA

GTTATGAGGACAGCACTTTTAACTACTTCCCATGGTACCAGCAGTTCCCTGGGGAAGGCCCTGCACTCCTGATATCC

ATACGTTCAGTGTCCGATAAAAAGGAAGATGGACGATTCACAATCTTCTTCAATAAAAGGGAGAAAAAGCTCTCCTT

GCACATCACAGACTCTCAGCCTGGAGACTCAGCTACCTACTTCTGTCTCAGCACGGGTGACAACTATCAGCTGATCT

GGGGCTCTGGGACCAAGCTAATTATAAAGCCAGACATCCAGAACCCAGAACCTGCTGTGTACCAGTTAAAAGATCCT

CGGTCTCAGGACAGCACCCTCTGCCTGTTCACCGACTTTGACTCCCAAATCAATGTGCCGAAAACCATGGAATCTGG

AACGTTCATCACTGACAAAACTGTGCTGGACATGAAAGCTATGGATTCCAAGAGCAATGGGGCCATTGCCTGGAGCA

ACCAGACAAGCTTCACCTGCCAAGATATCTTCAAAGAGACCAACGCCACCTACCCCAGTTCAGACGTTCCCTGTGAT

GCCACGTTGACCGAGAAAAGCTTTGAAACAGATATGAACCTAAACTTTCAAAACCTGTCAGTTATGGGACTCCGAAT

CCTCCTGCTGAAAGTAGCGGGATTTAACCTGCTCATGACGCTGAGGCTGTGGTCCGGCTCCGGAGCCACGAACTTCT

CTCTGTTAAAGCAAGCAGGAGACGTGGAAGAAAACCCCGGTCCCATGGCCCCCAGGCTCCTTTTCTGTCTGGTTCTT

TGCTTCTTGAGAGCAGAACCAACAAATGCTGGTGTCATCCAAACACCTAGGCACAAGGTGACAGGGAAGGGACAAGA

AGCAACTCTGTGGTGTGAGCCAATTTCAGGACATAGTGCTGTTTTCTGGTACAGACAGACCATTGTGCAGGGCCTGG

AGTTCCTGACTTACTTTCGAAATCAAGCTCCTATAGATGATTCAGGGATGCCCAAGGAACGATTCTCAGCTCAGATG

CCCAATCAGTCGCACTCAACTCTGAAGATCCAGAGCACGCAACCCCAGGACTCAGCGGTGTATCTTTGTGCAAGCAG

CTTAAGGGACAGGGTTCAAAACACCTTGTACTTTGGTGCGGGCACCCGACTATCGGTGCTAGAGGATCTGAGAAATG

TGACTCCACCCAAGGTCTCCTTGTTTGAGCCATCAAAAGCAGAGATTGCAAACAAACAAAAGGCTACCCTCGTGTGC

TTGGCCAGGGGCTTCTTCCCTGACCACGTGGAGCTGAGCTGGTGGGTGAATGGCAAGGAGGTCCACAGTGGGGTCAG

CACGGACCCTCAGGCCTACAAGGAGAGCAATTATAGCTACTGCCTGAGCAGCCGCCTGAGGGTCTCTGCTACCTTCT

GGCACAATCCTCGCAACCACTTCCGCTGCCAAGTGCAGTTCCATGGGCTTTCAGAGGAGGACAAGTGGCCAGAGGGC

TCACCCAAACCTGTCACACAGAACATCAGTGCAGAGGCCTGGGGCCGAGCAGACTGTGGGATTACCTCAGCATCCTA

TCAACAAGGGGTCTTGTCTGCCACCATCCTCTATGAGATCCTGCTAGGGAAAGCCACCCTGTATGCTGTGCTTGTCA

GTACACTGGTGGTGATGGCTATGGTCAAAAGAAAGAATTCATGA

TCR4

ATGGACAAGATCCTGACAGCAACGTTTTTACTCCTAGGCCTTCACCTAGCTGGGGTGAATGGCCAGCAGCAGGAGAA

ACGTGACCAGCAGCAGGTGAGACAAAGTCCCCAATCTCTGACAGTCTGGGAAGGAGAGACCGCAATTCTGAACTGCA

GTTATGAGGACAGCACTTTTAACTACTTCCCATGGTACCAGCAGTTCCCTGGGGAAGGCCCTGCACTCCTGATATCC

ATACGTTCAGTGTCCGATAAAAAGGAAGATGGACGATTCACAATCTTCTTCAATAAAAGGGAGAAAAAGCTCTCCTT

GCACATCACAGACTCTCAGCCTGGAGACTCAGCTACCTACTTCTGTGCAGCAAGGGAAGGTGACAACTATCAGCTGA

TCTGGGGCTCTGGGACCAAGCTAATTATAAAGCCAGACATCCAGAACCCAGAACCTGCTGTGTACCAGTTAAAAGAT

CCTCGGTCTCAGGACAGCACCCTCTGCCTGTTCACCGACTTTGACTCCCAAATCAATGTGCCGAAAACCATGGAATC

TGGAACGTTCATCACTGACAAAACTGTGCTGGACATGAAAGCTATGGATTCCAAGAGCAATGGGGCCATTGCCTGGA

GCAACCAGACAAGCTTCACCTGCCAAGATATCTTCAAAGAGACCAACGCCACCTACCCCAGTTCAGACGTTCCCTGT

GATGCCACGTTGACCGAGAAAAGCTTTGAAACAGATATGAACCTAAACTTTCAAAACCTGTCAGTTATGGGACTCCG

AATCCTCCTGCTGAAAGTAGCGGGATTTAACCTGCTCATGACGCTGAGGCTGTGGTCCGGCTCCGGAGCCACGAACT

TCTCTCTGTTAAAGCAAGCAGGAGACGTGGAAGAAAACCCCGGTCCCATGTCTAACACTGTCCTCGCTGATTCTGCC

TGGGGCATCACCCTGCTATCTTGGGTTACTGTCTTTCTCTTGGGAACAAGTTCAGCAGATTCTGGGGTTGTCCAGTC

TCCAAGACACATAATCAAAGAAAAGGGAGGAAGGTCCGTTCTGACGTGTATTCCCATCTCTGGACATAGCAATGTGG

TCTGGTACCAGCAGACTCTGGGGAAGGAATTAAAGTTCCTTATTCAGCATTATGAAAAGGTGGAGAGAGACAAAGGA

TTCCTACCCAGCAGATTCTCAGTCCAACAGTTTGATGACTATCACTCTGAAATGAACATGAGTGCCTTGGAACTGGA

GGACTCTGCTATGTACTTCTGTGCCAGCTCTCTCAGGCCTGGGGGGGTTGAACAGTACTTCGGTCCCGGCACCAGGC

TCACGGTTTTAGAGGATCTGAGAAATGTGACTCCACCCAAGGTCTCCTTGTTTGAGCCATCAAAAGCAGAGATTGCA

AACAAACAAAAGGCTACCCTCGTGTGCTTGGCCAGGGGCTTCTTCCCTGACCACGTGGAGCTGAGCTGGTGGGTGAA

TGGCAAGGAGGTCCACAGTGGGGTCAGCACGGACCCTCAGGCCTACAAGGAGAGCAATTATAGCTACTGCCTGAGCA

GCCGCCTGAGGGTCTCTGCTACCTTCTGGCACAATCCTCGCAACCACTTCCGCTGCCAAGTGCAGTTCCATGGGCTT

TCAGAGGAGGACAAGTGGCCAGAGGGCTCACCCAAACCTGTCACACAGAACATCAGTGCAGAGGCCTGGGGCCGAGC

AGACTGTGGGATTACCTCAGCATCCTATCAACAAGGGGTCTTGTCTGCCACCATCCTCTATGAGATCCTGCTAGGGA

AAGCCACCCTGTATGCTGTGCTTGTCAGTACACTGGTGGTGATGGCTATGGTCAAAAGAAAGAATTCATGA
