# Supplementary material for: Correction: Human and Murine Clonal CD8+ T Cell Expansions Arise during Tuberculosis Because of TCR Selection
Source: PLoS Pathog. 2015 Sep 17;11(9):e1005144. doi: 10.1371/journal.ppat.1005144 (PMC4574556; doi:10.1371/journal.ppat.1005144)
Supplement: S1 File — (PDF) [file ppat.1005144.s001.pdf]

PLOS | PATHOGENS

RESEARCH ARTICLE

Human and Murine Clonal CD8+ T Cell Expansions Arise during Tuberculosis Because of TCR Selection

Cláudio Nunes-Alves[1,2,3], Matthew G. Booty[1,4], Stephen M. Carpenter[1,5,6], Alissa C. Rothchild[1,4], Constance J. Martin[7], Danielle Desjardins[5], Katherine Steblenko[1], Henrik N. Kløverpris[8,9,10], Rajhmun Madansein[9], Duran Ramsuran[8], Alasdair Leslie[8,9], Margarida Correia-Neves[2,3], Samuel M. Behar[1]*

1 Department of Microbiology and Physiological Systems, University of Massachusetts Medical School, Worcester, Massachusetts, United States of America, **2** Life and Health Sciences Research Institute (ICVS), School of Health Sciences, University of Minho, Braga, Portugal, **3** ICVS/3B's—PT Government Associate Laboratory, Braga/Guimarães, Portugal, **4** Program in Immunology, Harvard Medical School, Boston, Massachusetts, United States of America, **5** Division of Infectious Disease, Department of Medicine, Brigham and Women's Hospital, Boston, Massachusetts, United States of America, **6** Division of Infectious Disease, Department of Medicine, University of Massachusetts Medical School, Worcester, Massachusetts, United States of America, **7** Department of Immunology and Infectious Diseases, Harvard T.H. Chan School of Public Health, Boston, Massachusetts, United States of America, **8** KwaZulu-Natal Research Institute for TB and HIV, Durban, South Africa, **9** Nelson Mandela School of Medicine, University of Kwa-Zulu-Natal, Durban, South Africa, **10** Department of International Health, Immunology and Microbiology, University of Copenhagen, Copenhagen, Denmark

* samuel.behar@umassmed.edu

CrossMark
click for updates

OPEN ACCESS

Citation: Nunes-Alves C, Booty MG, Carpenter SM, Rothchild AC, Martin CJ, Desjardins D, et al. (2015) Human and Murine Clonal CD8+ T Cell Expansions Arise during Tuberculosis Because of TCR Selection. PLoS Pathog 11(5): e1004849. doi:10.1371/journal.ppat.1004849

Editor: David M. Lewinsohn, Portland VA Medical Center, Oregon Health and Science University, UNITED STATES

Received: January 11, 2015

Accepted: April 1, 2015

Published: May 6, 2015

Copyright: © 2015 Nunes-Alves et al. This is an open access article distributed under the terms of the Creative Commons Attribution License, which permits unrestricted use, distribution, and reproduction in any medium, provided the original author and source are credited.

Data Availability Statement: The data files for this study are publicly deposited in the University of Massachusetts Medical School's institutional repository, eScholarship@UMMS. The permanent link to the data is http://dx.doi.org/10.13028/M2WC7N.

Funding: This work was supported by the Portuguese Foundation for Science and Technology individual fellowship (CNA) www.fct.pt, a National Institutes of Health Grant R01 AI106725 (SMB) www.nih.gov, and a Center for AIDS Research Grant P30

Abstract

The immune system can recognize virtually any antigen, yet T cell responses against several pathogens, including *Mycobacterium tuberculosis*, are restricted to a limited number of immunodominant epitopes. The host factors that affect immunodominance are incompletely understood. Whether immunodominant epitopes elicit protective CD8+ T cell responses or instead act as decoys to subvert immunity and allow pathogens to establish chronic infection is unknown. Here we show that anatomically distinct human granulomas contain clonally expanded CD8+ T cells with overlapping T cell receptor (TCR) repertoires. Similarly, the murine CD8+ T cell response against *M*. *tuberculosis* is dominated by TB10.44-11-specific T cells with extreme TCRβ bias. Using a retrogenic model of TB10.44-11-specific CD8+ T cells, we show that TCR dominance can arise because of competition between clonotypes driven by differences in affinity. Finally, we demonstrate that TB10.4-specific CD8+ T cells mediate protection against tuberculosis, which requires interferon-γ production and TAP1-dependent antigen presentation in vivo. Our study of how immunodominance, biased TCR repertoires, and protection are inter-related, provides a new way to measure the quality of T cell immunity, which if applied to vaccine evaluation, could enhance our understanding of how to elicit protective T cell immunity.

AI 060354 (SMB) www.nih.gov. The funders had no role in study design, data collection and analysis, decision to publish, or preparation of the manuscript.

Competing Interests: The authors have declared that no competing interests exist.

Author Summary

While T cells are required for protection against *Mycobacterium tuberculosis* infection, attempts to prevent tuberculosis by vaccines designed to elicit memory T cells have only been partially successful. Several vaccine candidates are in clinical trials, but progress has been slow because their ability to prevent disease must be empirically tested. There is little understanding of why certain antigens are targets of protective immunity. We have characterized an immunodominant $CD8^+$ T cell response to the *M. tuberculosis* antigen TB10.4 (EsxH). $CD8^+$ T cells specific for the $TB10.4_{4-11}$ epitope are primed early during infection and account for 30–50% of lung $CD8^+$ T cells during chronic infection. Now we have used deep sequencing to characterize the TCR repertoire of $TB10.4_{4\text{-}11}$-specific $CD8^+$ T cells in the lungs of infected mice. Interestingly, $TB10.4_{4\text{-}11}$-specific $CD8^+$ T cells exhibit extreme clonal expansion of certain TCRβ with common structural features, most likely because of affinity selection. Affinity selection of T cells is more important when antigen presentation is limiting. Although the lung contains numerous bacteria during infection, antigen-presentation by infected APC may be limiting, mimicking a "low antigen" state. Thus, even T cells that have the potential to mediate protection may function inefficiently because of suboptimal T cell activation.

Introduction

The adaptive immune system can generate 10^{14} unique TCRs, which provides the capacity to recognize an enormous universe of distinct antigens [1–4]. Despite our understanding of the genetic and structural basis for TCR diversity and antigen recognition, it remains challenging to predict the magnitude and diversity of T cell responses. The size of the T cell response to model antigens generally correlates with the abundance of antigen-specific T cells in the naïve repertoire (e.g., precursor frequency) [5–7]. Paradoxically, pathogen-specific T cell responses are often focused on a small number of the available antigenic epitopes and use a narrow TCR repertoire, a phenomenon termed "immunodominance". Pathogens have numerous strategies to evade host immunity, hindering our ability to determine a priori how T cell diversity relates to antimicrobial immunity. Thus, the relationship between immunodominance and host defense during infection is incompletely understood. For pathogens that rapidly mutate, such as human immunodeficiency virus 1 (HIV-1), a diverse T cell response could benefit the host by efficiently detecting escape mutants, while a biased response could be detrimental. For slowly replicating pathogens that encode numerous antigens, the relation between diversity and protection is less clear.

The *M. tuberculosis* genome contains hundreds of epitopes that can potentially be recognized by murine and human $CD8^+$ T cells [8]. The $CD8^+$ T cell response against *M. tuberculosis* focuses on the TB10.4 protein (EsxH; Rv0288) in people as well as experimentally infected animals [8–13]. Following aerosol infection of C57BL/6 mice, 30–50% of the responding $CD8^+$ T cells in the lungs recognize the K^b-restricted epitope $TB10.4_{4-11}$ (amino acid sequence IMYNYPAM), defining it as an immunodominant epitope [14–16]. Immunodominant T cell responses in patients with tuberculosis have been suggested to be both a correlate of protection and a marker of disease progression [17–20]. Elucidating how immunodominance arises and affects resistance to infection is crucial for developing successful vaccines, which usually target a limited number of antigens.

Here, we investigated the origin and protective capacity of immunodominant T cell responses following *M. tuberculosis* infection in both humans and mice. Extreme TCR bias, the

Table 1. Clinical characteristics of subjects.

case	TB Dx	Rx initiated	Resection	MDR XDR	HIV status	samples
0021	02/2003	9/9/12	6/24/14	Yes No	positive on ART	extensive disease and evidence of mycetoma
0023	03/2012	9/15/12	6/17/14	Yes unknown	positive on ART	A-Right upper lobe (RUL)- purulent, extensive cavities B-Right middle lobe (RML)- no cavities but evidence of disease C-Right lower lobe (RLL), least involved but still evidence of disease
0024	unknown	6/15/13	6/9/14	Yes unknown	negative	A-RUL, extensive cavities B-RML, no cavities but evidence of disease C-RLL least involved but still evidence of disease
0026	12/2012	5/10/14	5/27/14	No No	positive on ART	A LUL- extensive cavities and calcified lesions B LLL- apparently healthy tissue
0027	06/2012	1/15/14	6/3/14	No No	positive on ART	A RUL—Diseased tissue, cavities throughout B RML—No cavities but evidence of involvement

TB, tuberculosis; Dx, diagnosis; Rx, treatment; MDR, multidrug-resistant TB; XDR, extensively drug-resistant TB; ART, anti-retroviral therapy.

doi:10.1371/journal.ppat.1004849.t001

presence of public TCRs, and strong selection of a complementarity determining region 3 (CDR3) β motif were shown by TCR sequencing of sorted tetramer$^+$ cells from the lungs of infected mice. We discovered that TCR bias emerges soon after T cell priming in the lymph node (LN) and becomes more extreme during chronic infection. Cloning TB10.4$_{4-11}$-specific TCRs allowed us to develop retrogenic (Rg) mice to study immunodominant TCRs in vivo. Competition studies using TB10.4$_{4-11}$-specific Rg CD8$^+$ T cells showed that small differences in T cell affinity lead to clonal dominance in vivo. Finally, TB10.4$_{4-11}$-specific CD8$^+$ T cells transferred IFNγ-dependent protection against *M. tuberculosis* infection that required TAP1-dependent antigen presentation.

Results

Human CD8$^+$ T cell expansions in lung granulomas

Deep sequencing of the TCRβ repertoire was performed on CD8$^+$ T cells purified from 11 lung granulomas and one LN obtained from 5 patients undergoing lung resection for medically non-responsive tuberculosis (see Table 1, S1 Data). The CD8$^+$ T cells in the granulomas were more clonal than the peripheral blood TCRβ repertoire of healthy individuals (Fig 1A and S2A Data). TCRβ expansions were detected in all lung samples (Fig 1B and S3 Data). Abundant clonotypes were detected in anatomically distinct granulomas from the same patient (Fig 1B). There was extensive overlap of the TCR sequences between granulomas from the same patient, although most sequences detected in each granuloma were unique to that lesion (Fig 1C). However, it was the shared sequences that were most abundant. For example, of 217 TCRβs common to all three granulomas from patient #23, 143 were the most abundant in each lesion (the top 10 are shown in Fig 1C and S2B Data). Thus, the same T cells clonotypes were abundant in different lesions from the same subject despite their varied pathology (see Table 1). Some TCRβs appeared to have undergone antigen-selection, as sequences with identical CDR3β amino acid sequences were encoded by distinct recombination events. For example, five distinct DNA recombination events led to the CDR3β sequence 'CASSVDGGTEAFF' and two occurred at a high frequency (1.16% and 0.9%, Fig 1D). Thus, CD8$^+$ T cells in human granulomas undergo clonal expansion, and while there is considerable heterogeneity between distinct lesions, the most abundant clonotypes were shared between granulomas. As the antigen-specificity and the HLA restriction of these T cells were unknown, the inferences that we can make are limited. Furthermore, co-infection with HIV, present in four of the five subjects,

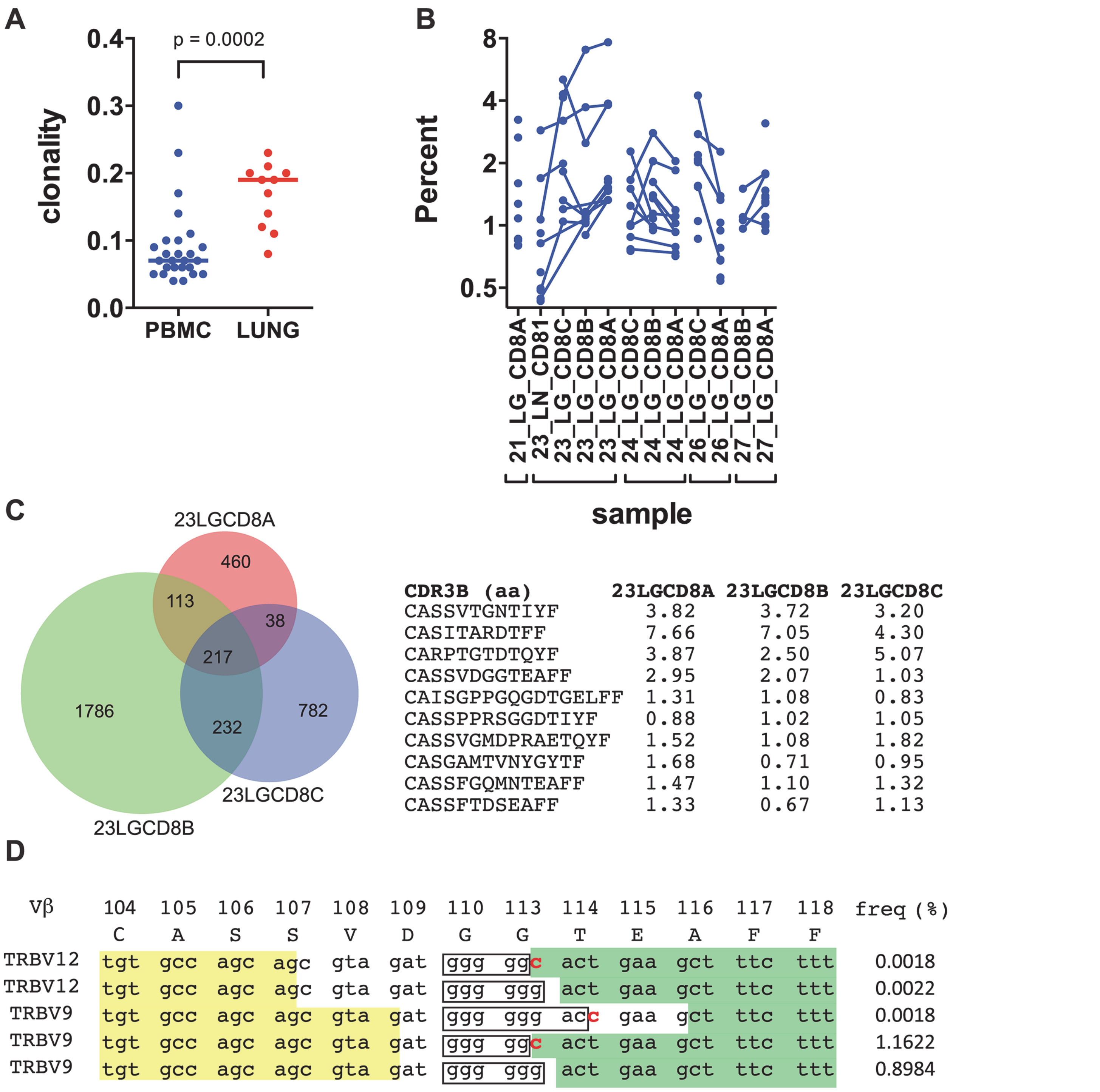

Fig 1. Human CD8+ T cells undergo clonal expansions in lung granulomas. (**a**) Clonality of CD8+ TCR sequences obtained from pulmonary granulomas (n = 11) compared to PBMC from healthy donors (n = 26); p = 0.0002 by Mann-Whitney. (**b**) The frequency of the top ten clones in each sample. Clones shared between samples are connected with a line. (**c**) TCRVβ chains are shared among anatomically distinct granulomas from the same patient. A Venn diagram shows the overlap of unique TCRs from samples 23A, 23B, and 23C. The CDR3β amino acid sequences of the 10 most frequent clones that are shared by the 3 lesions are shown, along with their respective frequency in each lesion. (**d**) TCR selection suggested by distinct DNA sequences that encode the same CDR3β amino acid sequence. Sample 23LGCD8B contains five distinct recombination events encode the CDR3β amino acid sequence "CASSVDGGTEAFF." Yellow shading, 3' Vβ gene sequence; boxed sequence, D nucleotides; green shading, 5' Jβ gene sequence; bold red letters indicate differences in the sequences. Other differences exist in the Vβ gene sequences. The respective frequency of each sequence is shown.

doi:10.1371/journal.ppat.1004849.g001

could potentially confound the analysis, since HIV itself can alter the TCR repertoire of $CD8^+$ T cells. Indeed, we cannot be certain whether these TCRs are specific for Mtb. Therefore, we next turned to an animal model of tuberculosis to address the origin and consequences of clonal $CD8^+$ T cell expansions.

The TB10.4-specific CD8+ T cell response is highly biased

The *M*. *tuberculosis* epitope $TB10.4_{4-11}$ elicits an immunodominant $CD8^+$ T cell response in both people and mice [10,21]. To determine whether the number of distinct clonotypes among the responding T cells was limited or diverse, we sorted $TB10.4_{4-11}$-$tetramer^+CD8^+$ T cells from the lungs of six individual mice, infected with *M*. *tuberculosis* for nine weeks. Deep sequencing showed that the $CD8^+$ T cell response to $TB10.4_{4-11}$ was significantly more clonal than the T cell repertoire from uninfected mice (Fig 2A). The TCRβ repertoire of each infected mouse was dominated by large expansions of two or three clones, although the dominant Vβ gene varied between individuals (Fig 2B). The dominance of TCRβ chains with identical CDR3β sequences indicated that the $TB10.4_{4-11}$-specific $CD8^+$ T cell response was oligoclonal (Fig 2C).

To determine whether TCR bias was established during priming in the LN or after T cell trafficking to the lungs, we used monoclonal antibodies (mAbs) specific for a subset of the known Vβ families (see Table 2). All 5 Vβ families were expressed by $CD8^+$ T cells obtained from LNs of mice 21 days after infection, the majority of which are not specific for Mtb (Fig 2D). The distribution of these 5 Vβ families among tetramer+ cells in the LN was similar to the bulk $CD8^+$ T cell population (Fig 2D). Starting on day 21 in the lung, and more dramatically by day 28 in the LN and lung, significant Vβ family bias was detected among $TB10.4_{4-11}$-specific $CD8^+$ T cells. For example, Vβ4 (mouse 'A' and 'B'), Vβ7 (mouse 'B'), or Vβ10 (mouse 'C') dominated the $TB10.4_{4-11}$-specific $CD8^+$ T cell response by day 21 post infection (Fig 2E). The dominant Vβ family was frequently expanded in both the LN and lung, suggesting that TCR bias developed early after T cell priming. Overtime, biases became more dramatic, suggesting ongoing preferential expansion of certain clones. The $TB10.4_{4-11}$-specific $CD8^+$ T cells in one infected mouse (mouse 'H') were >80% $V\beta7^+$ after 25 weeks of infection (Fig 2E and 2'H'). Thus, after an initial priming of a broad repertoire in response to antigen, TCR bias among $TB10.4_{4-11}$-specific $CD8^+$ T cells develops in the draining LN early after T cell priming and becomes established during the chronic phase of infection. The highly oligoclonal response and the dominance of CDR3β amino acid sequences suggest that the immunodominant T cell clonotypes undergo selection during infection.

The paradox of high precursor frequency and extreme TCR bias

Two general mechanisms can explain how TCR bias develops during the $TB10.4_{4-11}$ response: 1) $TB10.4_{4-11}$-specific $CD8^+$ T cells are drawn from a limited pool of naïve precursors; or 2) the naïve $TB10.4_{4-11}$-specific $CD8^+$ T cells pool is diverse but competition leads to selection and bias. To discriminate between these possibilities, we measured the precursor frequency of $TB10.4_{4-11}$-specific $CD8^+$ T cells in uninfected mice.

Using sequential tetramer staining and enrichment of antigen-specific T cells from the naïve repertoire [5,22], we determined that approximately 1:13,000 $CD8^+$ T cells were specific for the $TB10.4_{4-11}$ epitope in the C57BL/6 naïve repertoire, or about 857 cells per mouse (Fig 3A and 3B). This precursor frequency is among the highest recorded for antigen-specific $CD8^+$ T cells in the mouse [7].

The high frequency of $TB10.4_{4-11}$-specific $CD8^+$ T cell precursors in C57BL/6 mice contrasts with the limited number of unique T cell clonotypes that comprise the post-infection

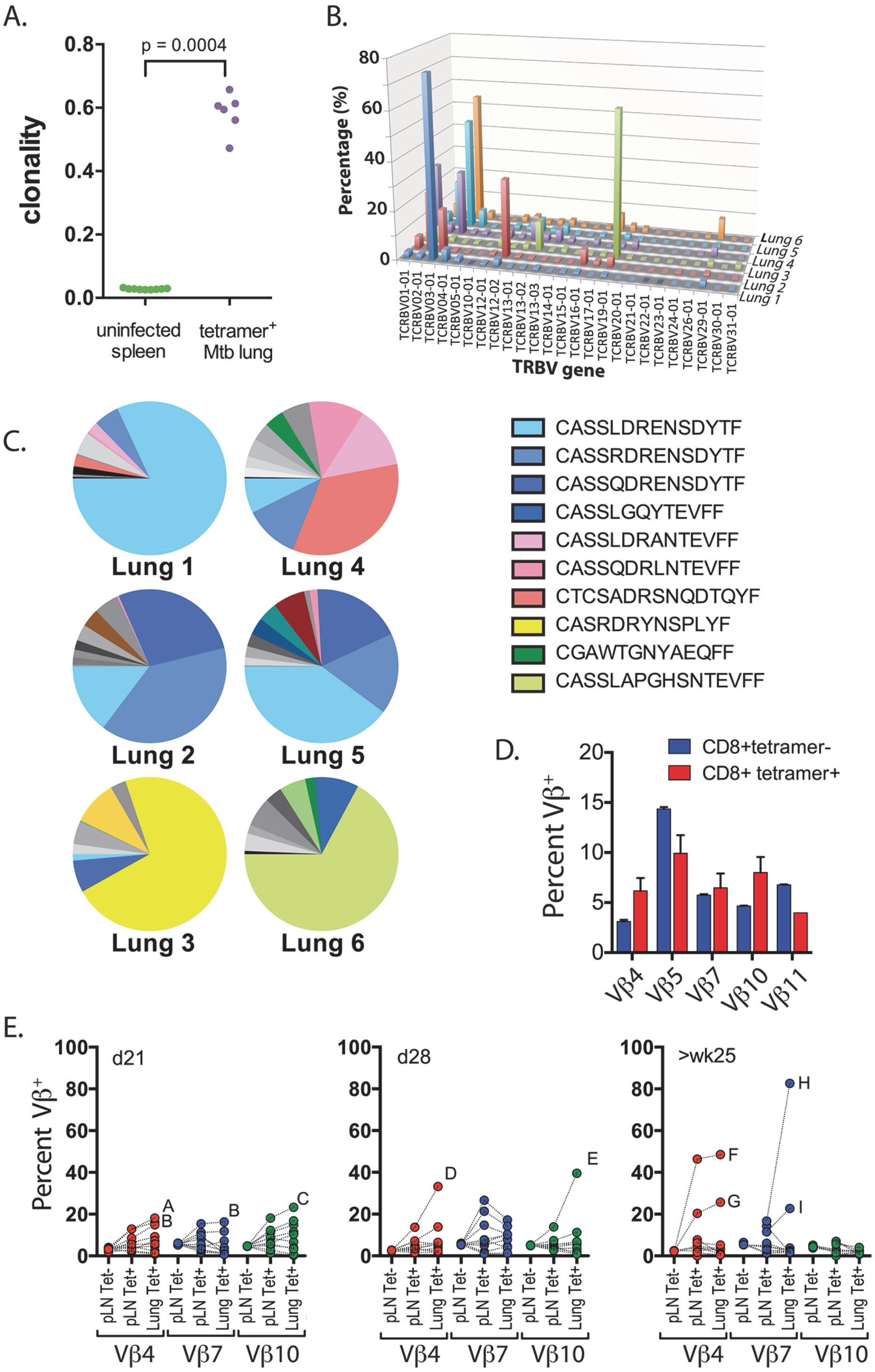

Fig 2. Clonal expansions of CD8+ T cells specific for the immunodominant antigen TB10.4. (**a**) Clonality of CD8+ TCR sequences obtained from infected lung granulomas (n = 6) compared to splenocytes from uninfected mice (n = 9); p = 0.0004 by Mann-Whitney. (**b,c**) Frequency of TCR Vβ families (**b**) and CDR3βs (**c**) of $TB10.4_{4-11}$-specific $CD8^+$ T cells from the lungs of C57BL/6J mice infected with *M. tuberculosis* for 9 weeks. Data were obtained by Next-generation sequencing of tetramer-purified T cells from 6 individual mice. (**d**) Frequency of TCR Vβ4 (TRBV2), Vβ5 (TRBV12), Vβ7 (TRBV29), Vβ10 (TRBV4) and Vβ11 (TRBV16) families of $TB10.4_{4-11}$-tetramer$^+$CD8$^+$T (red) or $TB10.4_{4-11}$-tetramer$^-$CD8$^+$T (blue) cells from the pulmonary LN of C57BL/6J mice infected with *M. tuberculosis* at 21 days post infection. (**e**) Frequency of TCR Vβ4 (TRBV2), Vβ7 (TRBV29) and Vβ10 (TRBV4) families of $CD8^+$ T cells from the pulmonary LN or

lung of C57BL/6J mice infected with *M. tuberculosis* at 21 days (left panel), 28 days (middle panel) or >25 weeks (right panel) post infection. Dotted lines connect data from individual mice, some of which are labeled (A through I) to highlight mice with biased Vβ family usage. Data were obtained by flow cytometric analysis of TB10.4$_{4-11}$-tetramer^{+}CD8^{+} T cells (Tet+) or TB10.4$_{4-11}$-tetramer^{-}CD8^{+} T cells (Tet-) from three independent experiments, each with 4–10 mice per group.

doi:10.1371/journal.ppat.1004849.g002

TB10.4$_{4-11}$-specific CD8^{+} T cell repertoire. To estimate the frequency of TB10.4$_{4-11}$-specific TCRβ clones in the naïve repertoire, TB10.4$_{4-11}$-specific sequences from infected mice were used to interrogate data sets from three uninfected C57BL/6 mice (referred to below as spleen A, B, or C) each containing more than a million TCRβ sequences (Provided by David Hamm, Adaptive Biotechnologies reference data "Mus musculus TCR Beta from spleen", 2014). By performing a pairwise comparison, we identified TB10.4$_{4-11}$-specific TCRβ DNA sequences in the naïve T cell repertoire of uninfected mice (Fig 3C). For example, an abundant TCRβ from the lung of infected mouse #2 (17.8%) was present in spleen B (0.0026%); however, none of the abundant (>0.1%) TCRβs from the lung of infected mouse #2 were present in spleen A or C (Fig 3C). When the translated CDR3β amino acid sequences were used to query the naïve repertoire, the number of matches increased from 40 to 345; and several of the most highly represented CDR3βs were identified (Fig 3D). This raises the possibility that there are multiple DNA recombination events that can generate CDR3β regions capable of recognizing TB10.4$_{4–11}$ in the naïve repertoire. Importantly, the frequencies of TB10.4$_{4–11}$–associated TCRβs in the naïve repertoire were similar to the median frequency determined for the entire naïve T cell population (Fig 3E). For example, the median frequency of the clonotypes detected in naïve spleen #B was 0.0011%. The frequencies of "CASSQDRENSDYTF" and "CASSRDRENSDYTF" in the naïve spleen #B repertoire were 0.00425% and 0.00106%, respectively. Thus, the low frequency of these two CDR3β regions in the naïve TCR repertoire does not predict their massive clonal expansion after infection.

To make this analysis more quantitative, we determined the frequencies of all TB10.4$_{4-11}$-associated CDR3β sequences that could be identified in any of the three uninfected spleens (A, B, or C). Those sequences ("TB10-associated", Fig 3E) had a slightly higher median frequency than the median of the entire splenic TCR repertoire ("uninfected spleen) (0.001845% vs. 0.001142%, $P < 0.0001$). We next focused on the subset of "TB10-associated" CDR3βs that were present in at least 2 of the 6 infected lungs, and defined these as "shared sequences" (Fig 3E). The median frequency of the "shared sequences" was 0.003614%, which was increased compared to "uninfected spleen" ($P < 0.0001$); this is in agreement with previous observations describing a higher frequency of public TCRs in the naïve repertoire [2]. Finally, the CDR3βs that we defined as "expanded sequences" (those with a frequency >1% among CD8+ T cells in the Mtb-infected lung and that account for 75% of the total TB10.4$_{4-11}$-associated sequences)

Table 2. Anti-TCR antibodies and their specificity.

mAb clone	Commercialdesignation	TRAV/TRBV gene family[1]
B20.1	Va2	TRAV14
KT4	Vb4	TRBV2
MR9-4	Vb5	TRBV12-1, TRBV12-2
TR310	Vb7	TRBV29
B21.5	Vb10	TRBV4
RR3-15	Vb11	TRBV16

[1] Based on data from http://www.imgt.org

doi:10.1371/journal.ppat.1004849.t002

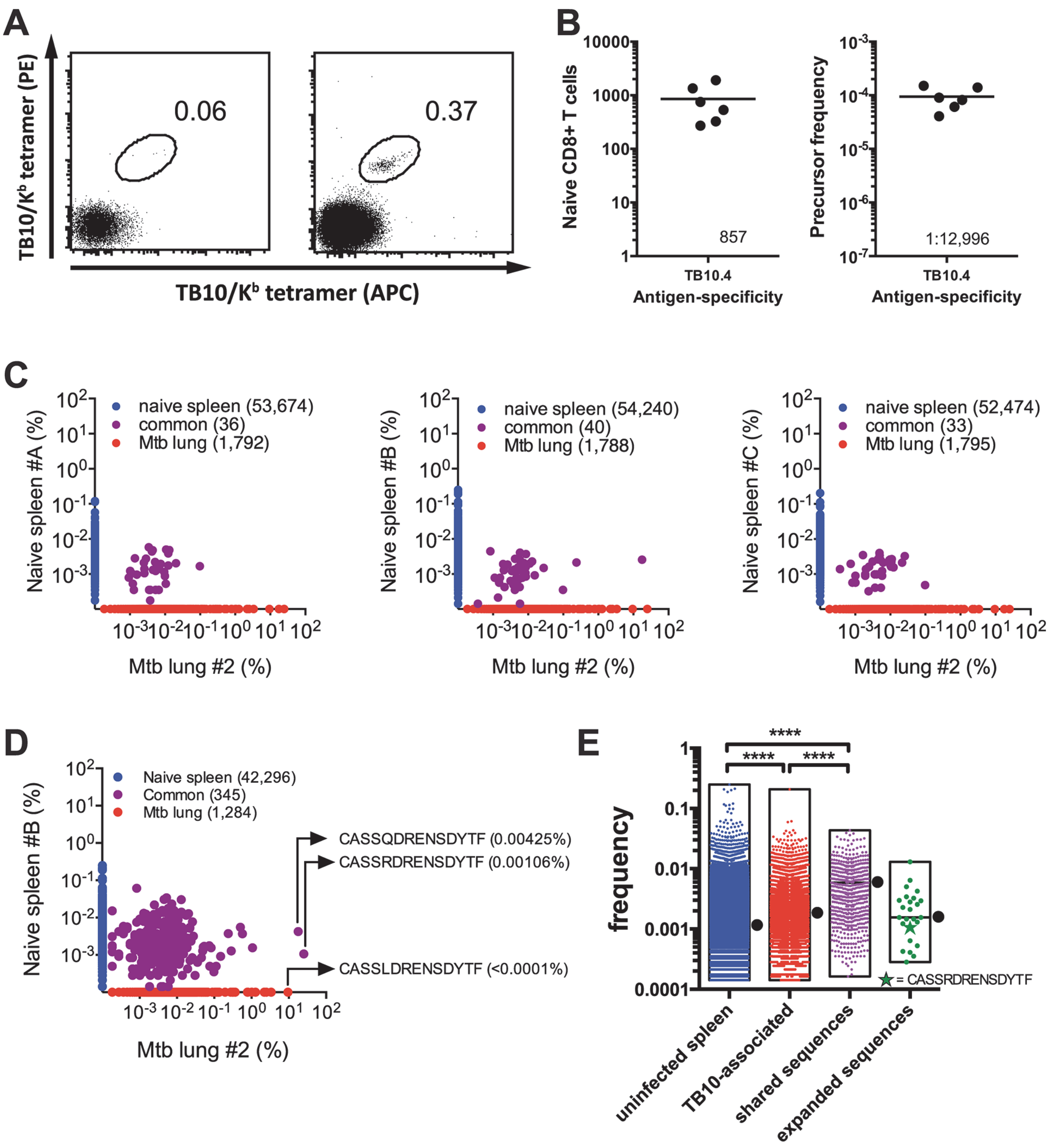

Fig 3. Precursor frequency of TB10.4-specific T cells. (**a,b**) Frequency of TB10.4$_{4-11}$-specific CD8$^+$ T cells in naïve C57BL/6J mice. (**a**) Represented are dot plots of dual tetramer staining in CD8$^+$ T cells in the unbound (left) or bound (right) fractions after immunomagnetic enrichment using tetramers. (**b**) Quantification of TB10.4$_{4-11}$-specific CD8$^+$ T cells in naïve C57BL/6J mice, either as the total number (left panel) or as the frequency among CD8$^+$ T cells (right panel). Data are representative from two experiments with 6–10 mice. (**c,d,e**) Frequency of TB10.4$_{4-11}$-associated TCRβ DNA (**c**) or amino acid (**d,e**) sequences in the T cell repertoire of uninfected C57BL/6J mice. (**c**) The unique TB10.4$_{4-11}$-associated TCRβ DNA sequences from lung#2 were compared pairwise to the TCRβ repertoire of three uninfected mice (spleen A-C). The frequency of unique TCRβ DNA sequences from uninfected mice are on the y-axes (blue); from *M*. *tuberculosis* infected lung on the x-axes (red); and sequences detected in both samples are colored purple. The number of unique sequences in each class is indicated in parentheses. (**d**) A pairwise comparison of unique TB10.4$_{4-11}$-associated CDR3β amino acid sequences from lung#2 and uninfected spleen B. (**e**) The frequency distribution of unique CDR3β amino acid sequences in the uninfected TCR repertoire. 'Uninfected' consists of all sequences from uninfected C57BL/6 mice. 'TB10-specific' are the TB10-specific sequences found in the uninfected splenic repertoire; 'shared sequences' are TB10-specific sequences detected in more than 2 infected mice; 'expanded sequences' are all "shared sequences" with frequency >1% in at least one infected mouse. Box represents minimum and maximum, and bar and dot is the median frequency. NS, not significant; ****, $p < 0.0001$, by one-way ANOVA with Kruskal-Wallis post test.

doi:10.1371/journal.ppat.1004849.g003

had a frequency similar to "uninfected spleen" (0.001559%, not significant). Thus, TB10.$4_{4\text{-}11}$-associated sequences do not appear to be over-represented in the naïve repertoire compared to other sequences.

Interestingly, the individual frequency of highly represented sequences within an individual naive mouse varied by more than 10-fold, raising the possibility that the precursor frequency of individual CDR3β clonotypes could affect their representation in the post-immune repertoire (e.g., after infection). Interestingly, "CASSRDRENSDYTF," which was one of the more successful CDR3βs as it was commonly detected during Mtb infection, was not present at a greater frequency than other TCRs in the naïve repertoire (Fig 3E).

Although T cells specific for the immunodominant epitope TB10.4_{4-11} have a high precursor frequency in the naïve repertoire of C57BL/6 mice (Fig 3B), extreme TCR bias emerges during the $CD8^+$ T cell response to TB10.4_{4-11} (Fig 2b). While heterogeneity in the frequency of TB10.$4_{4\text{-}11}$-specific clonotypes found in the naïve repertoire could account for some of the bias (Fig 3E), the precursor frequency of individual clonotypes in the naïve repertoire does not appear to be the dominant factor influencing representation in the post-infection repertoire. We next evaluated the possibility that clonotypic dominance occurs because of clonal selection during infection.

Evidence for antigen-driven peripheral T cell selection

The CDR3 length of the unique TCRβ sequences from the infected and uninfected mice was similar with a median CDR3β length of 36–39 bases (Fig 4A). In contrast, 56% of the highly represented TCRs (e.g., frequency >1%) had a CDR3β length of 42. Analysis of the amino acid sequences with this CDR3β length (accounting for 36% of all TB10.$4_{4\text{-}11}$-specific sequences) revealed a strong consensus motif—"CASSxDReNsdytF" (Fig 4B). This motif was present in clonal expansions if different mice, and several distinct DNA recombination events generate the conserved aspartic acid (Asp, "D") at CDR3β residue 6 (e.g., V-D recombination, germline encoded, N region addition; Fig 4C). Thus, we conclude that this residue is under strong selection.

In addition to the selection of the conserved Asp, the over-representation of certain CDR3β regions in their entirety appeared to be the consequence of selection. In Mouse #1, 63% of the CDR3β sequences encoded the amino acid sequence "CASSLDRENSDYTF." Remarkably, three distinct VDJ recombination events generated this CDR3β in Mouse #1 (Fig 4C). These data strongly suggest that these TCRs were selected. Not only were the TCRβ expansions extremely biased within each individual mouse, but some abundant sequences shared identical CDR3βs between mice, constituting so called "public" TCRs [2,23] (Fig 4C).

The "DREN" motif was also detected among TCRs from human lung granulomas in three of the five patients, and was expanded in patient #24 (Fig 4D). Two different patients share the motif "CASSxDRENTEAFF," which is similar to the murine motif (Fig 4D). While the "DREN" motif was detected in the peripheral blood TCRβ repertoire of normal donors, those clonotypes had a 10-fold lower average frequency (Fig 4D and S4 Data). Some clonotypes found in the Mtb granulomas were 1000-fold enriched compared to the average "DREN" frequency in peripheral blood of normal donors (Fig 4D). These data are consistent with selection since distinct recombination events generate different TCRs with the "DREN" motif (Fig 4D). Finally, there is evidence for public TCRs as patients #23 and #26 have clones with the identical CDR3β sequence "CASSSDRENTEAFF".

The extreme TCR bias (Fig 2C) and the identification of multiple VDJ recombination events encoding identical CDR3βs indicate that dominant T cell clonotypes undergo selective expansion during infection. To quantify selection during the response to *M. tuberculosis*, we

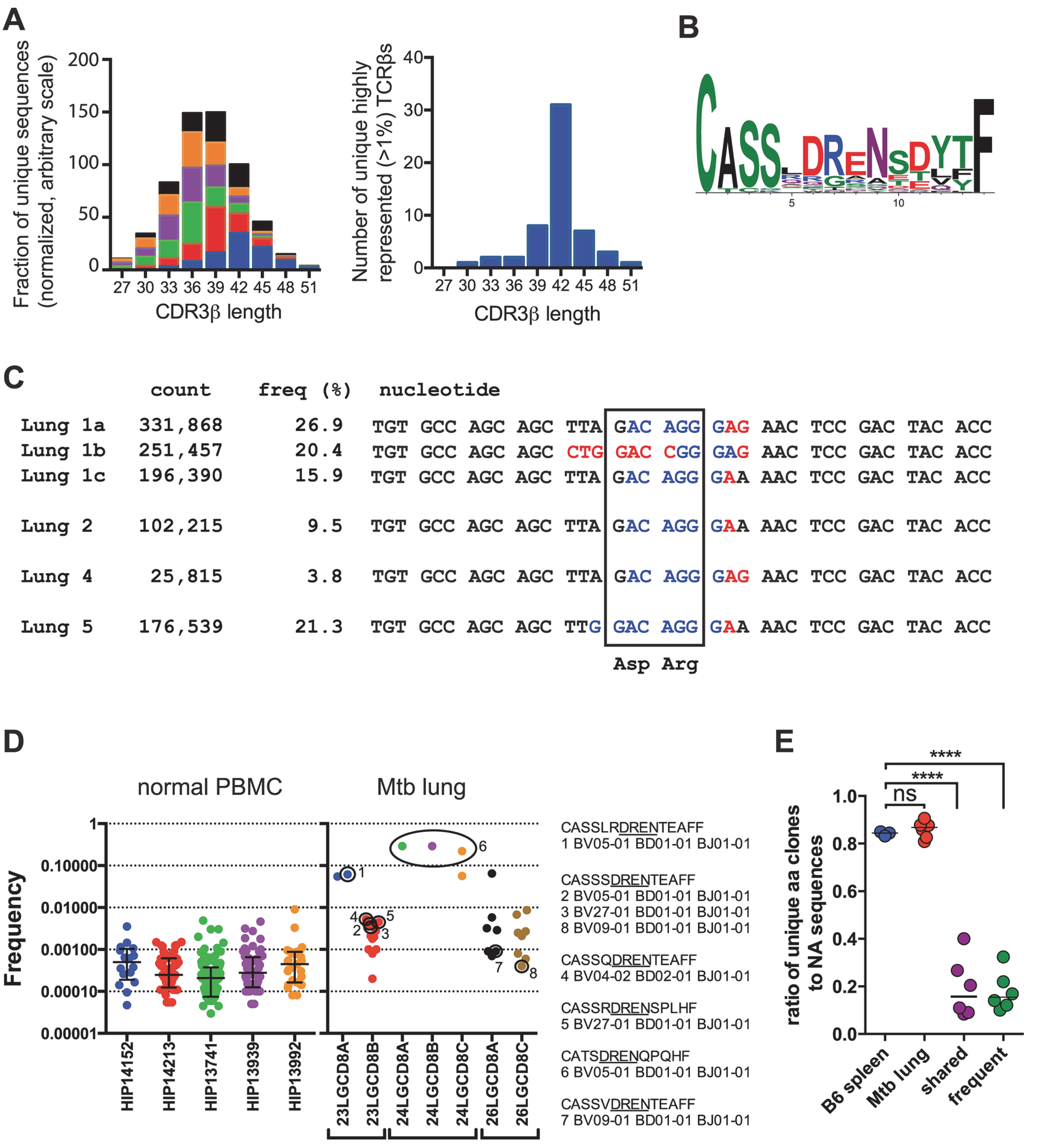

Fig 4. TB10.4-specific CD8+ T cells are selected during infection. (**a**) Frequency distribution of CDR3β amino acid length of TB10.$4_{4\text{-}11}$-specific CD8$^+$ T cells from the lungs of *M*. *tuberculosis* infected C57BL/6J mice. (**b**) Consensus analysis of the CDR3β amino acid sequence of TB10.$4_{4\text{-}11}$-specific CD8$^+$ T cells with 14 amino acids in length. (**c**) VDJ DNA rearrangements for the public CDR3β CASSLDRENSDYTF found in four different C57BL/6J mice, showing Vβ (black), N (red), Dβ (blue), and Jβ (black) sequences. The count and frequency for each sequence in the respective lung is also shown. The box highlights the nucleotides that encode the conserved aspartic acid (Asp, "D") and arginine (Arg, "R") residues. (**d**) Frequency of human TCRs containing the "DREN"

motif among normal PBMC or CD8$^+$ T cells from TB patients. Each point represents a unique clonotype and their corresponding CDR3β amino acid sequences are shown for some. (**e**) Ratio of unique amino acid clones to nucleotide sequences in T cells from naïve and infected C57BL/6J mice. TCR sequences were analyzed from uninfected 'B6 spleen' (n = 3 mice); infected 'Mtb lung' (TB10.4$_{4\text{-}11}$-specific CD8$^+$ T cells; n = 6 mice); or the following subsets of sequences: 'frequent' (>1% of the TB10-specific sequences) or 'shared sequences' (TB10.4$_{4\text{-}11}$-specific TCRs present in at least 2 mice). ****, p < 0.05 by one-way ANOVA and Holm-Sidak's multiple comparison test.

doi:10.1371/journal.ppat.1004849.g004

calculated the ratio of unique amino acid sequences to unique nucleic acid sequences. For example, the same immunodominant CDR3β was encoded by three unique DNA sequences in mouse #1. Shared sequences (>2 mice) and abundant sequences (>1%) had lower median values (0.158 and 0.154, respectively) than the bulk population of TB10.4$_{4\text{-}11}$-specific CD8$^+$ T cells (0.868) or T cells from uninfected mice (0.845), indicating strong selection at the level of the CDR3β amino acid sequence ($p < 0.0001$; Fig 4E). Thus, our TCR analysis indicates that clonotypic dominance occurs because of strong selection of certain CDR3β amino acid sequences.

Retrogenic mouse model for TB10.4-specific CD8+ T cells

To study the biology of TB10.4$_{4\text{-}11}$-specific CD8$^+$ T cells and to delineate the mechanism(s) responsible for the development of TCR bias during *M*. *tuberculosis* infection, we developed retrogenic mice. To ensure that we could track the recombinant TCR-expressing CD8$^+$ T cells, we used Vα2var mice, which have a single Vα gene (Vα2) but can still generate diversity through limited VαJα recombination [24]. Vα2var mice resisted tuberculosis and generated a dominant TB10.4$_{4\text{-}11}$-specific CD8$^+$ T cell response, although it was more variable than in C57BL/6 mice (see S5 Data).

Single cell sorting of H2-K^b/TB10.4$_{4–11}$ tetramer$^+$ cells from *M*. *tuberculosis*-infected Vα2var mice was followed by single cell PCR to determine the TCRα and TCRβ sequences. Three mice were analyzed and the TB10.4$_{4\text{-}11}$-specific CD8$^+$ T cell response in each mouse was dominated by one or two TCR clonotypes (Fig 5A). Each identified dominant TCRβ paired with a single TCRα chain, supporting that these were true clonal expansions. Despite the constrained Vα2, the VαJα recombination site was remarkably diverse. In contrast, there were structural parallels between the CDR3β sequences from Vα2var (Fig 5B) and C57BL/6 mice (Figs 2 and 3). While the dominant TCRβs from the Vα2var mice used distinct Vβs and Jβs, there was enrichment of arginine ("R") and aspartic acid ("D") in the CDR3β (Fig 5B and 5C). To determine whether the enrichment of "R" or "D" was significant, we assessed their occurrence in the normal TCRβ repertoire. We queried the splenic TCRβ repertoire from three C57BL/6 mice representing over 1.1 million reads and ~53,000 unique sequences each (Fig 2A). The average frequency of "R", "D", or "RD" at CDR3β position 6, 7, or 6–7, was 10.1%, 7.5%, and 2.5% respectively, indicating "R", "D", and "RD" were significantly enriched among the clonally expanded TB10.4$_{4\text{-}11}$-specific CD8$^+$ T cells ($P < 0.0001$; see S6 Data).

The four dominant TCRs were cloned into retroviral vectors linked by the 2A sequence allowing the production of four different retrogenic mice (named Rg1 to Rg4, see S10 Data) that expressed greater numbers of CD8$^+$ T cells specific for TB10.4$_{4–11}$ (Fig 5D). We confirmed that the correct TCRs were expressed based on expression of the expected Vα and Vβ chains by CD8$^+$ T cells from the retrogenic mice (Fig 5D). The four TCRs cloned were shown to be specific for TB10.4$_{4–11}$ based on their binding to tetramers and activation of effector functions following stimulation with the TB10.4$_{4–11}$ peptide (Fig 5E–5G).

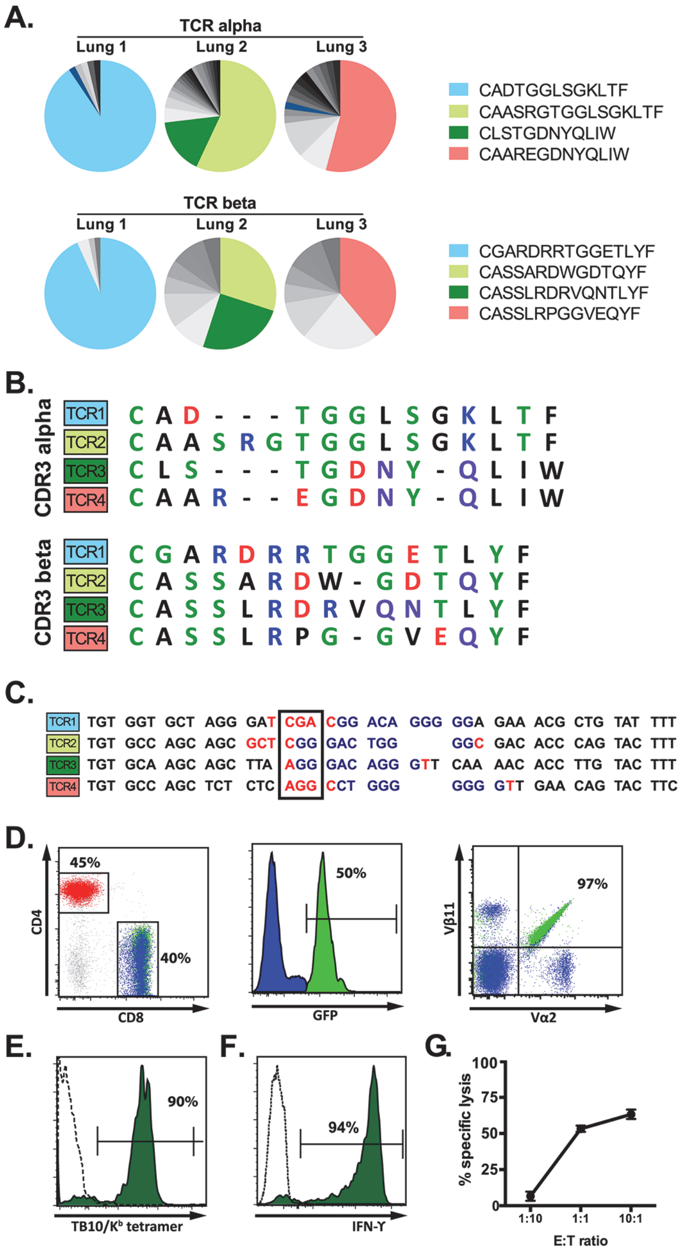

Fig 5. A retrogenic mouse model for TB10-specific CD8+ T cells. (**a**) Frequency and amino acid sequences of CDR3α (top panel) and CDR3β (bottom panel) regions of TB10.4$_{4-11}$-specific CD8$^+$ T cells from the lung of individual Vα2var mice infected with *M. tuberculosis*. Data were obtained by single-cell sorting and sequencing of 60–200 cells per mouse (n = 3). (**b**) Sequences of CDR3α (top panel) and CDR3β (bottom panel) amino acid sequences of the dominant clones of TB10.4$_{4-11}$-specific CD8$^+$ T cells expanded in the lung of Vα2var mice infected with *M. tuberculosis*. Dashes are gaps introduced for purpose of sequence alignment. (**c**) VDJ rearrangement (CDR3β) of the dominant TB10.4$_{4-11}$-specific CD8$^+$ clonotypes from the lung of Vα2var mice infected with *M. tuberculosis*, displaying Vβ (black), N (red), Dβ (blue), and Jβ (black) sequences. Boxed codon is the conserved arginine (Arg, "R"). (**d,e**) Flow-cytometry analysis of T cells from uninfected retrogenic mice expressing TCR3. (**d**) Represented are a dot plot of CD4 vs CD8 staining of CD3$^+$ T cells (left panel), histograms of GFP expression within CD8$^+$ T cells (middle panel), and a dot plot of Vα2 vs. Vβ11 staining in CD8$^+$ T cells (right panel). Red, CD4$^+$ T cells; blue, are colored in CD8$^+$GFP$^-$ T cells; green, CD8$^+$GFP$^+$ T cells. The numbers represent: the frequency of CD4$^+$ vs. CD8$^+$ T cells among gated CD3$^+$ T cells (left); the frequency of CD3$^+$CD8$^+$ cells that express GFP (middle); and the frequency of CD3$^+$CD8$^+$GFP$^+$ cells that are Vα2$^+$Vβ11$^+$ (right). **(e)** Tetramer staining of CD8$^+$GFP$^-$ T cells (dashed line) and CD8$^+$GFP$^+$ T cells (filled green histogram). The numbers represent the frequency of CD3$^+$CD8$^+$GFP$^+$ cells that are stained by the tetramer. (**f**) IFNγ production by lung T cells from TCR3 retrogenic mice infected

with *M. tuberculosis*. Lung cells were stimulated with TB10.4_{4-11}-peptide and analyzed by intracellular cytokine staining and flow cytometry. Represented are $CD8^{+}GFP^{+}$ T cells stimulated by TB10.4_{4-11}-peptide (filled green histogram) or left unstimulated (dashed line). The numbers represent the frequency of $CD3^{+}CD8^{+}GFP^{+}$ cells that produce IFNγ after peptide stimulation. (**g**) Specific lysis of TB10.4_{4-11}-peptide pulsed EL4 targets by TCR3 retrogenic T cells at different effector to target ratios. Data are representative of >10 (d, e), >5 (f), and 3 (g) independent experiments.

doi:10.1371/journal.ppat.1004849.g005

Priming and acquisition of effector function by naïve TB10.44-11-specific CD8+ T cells

Naïve ($CD44^{lo}CD62L^{hi}$) Rg3 $CD8^{+}$ T cells were purified and transferred into congenically marked recipient mice infected with *M. tuberculosis*. Immediately after transfer (d6 or d7 after infection) very few Rg $CD8^{+}$ T cells were detected (~100–200 cells per lung) and they were mostly naïve (Fig 6A). Rg $CD8^{+}$ T cells began to acquire an activated phenotype ($CD44^{hi}CD62L^{lo}$) starting on d11 in the LN. Associated with their activation, the numbers of Rg3 $CD8^{+}$ T cells in the LN dramatically increased during days 11–13 post-infection (Fig 6A). Subsequently, these Rg $CD8^{+}$ T cells began to accumulate in the lung by day 13–15, and the Rg3 $CD8^{+}$ T cell numbers continued to increase through day 18 (Fig 6A). The increase in cell numbers in the LN and lung correlated with proliferation, occurring first in the LN, then in the lung, and finally in the spleen (Fig 6B). Thus, priming of Rg3 $CD8^{+}$ T cells tracks the kinetics that has been established for the IFNγ-response in intact mice and activation of transferred transgenic $CD4^{+}$ T cells [25–28]. Following priming, the Rg3 $CD8^{+}$ T cells rapidly acquired the ability to produce IFNγ in both the LN and lung (Fig 6C). Thus, Rg3 $CD8^{+}$ T cells are primed in the LN and are subsequently recruited to the lung, where they express a variety of effector functions including the production of IFNγ, TNF, and granzyme B (see S7 Data), similar to endogenous TB10.4_{4-11}-specific $CD8^{+}$ T cells.

TAP1 and IFNγ are required for protection mediated by CD8+ T cells

It is unknown whether the immunodominant $CD8^{+}$ T cell response to TB10.4 is protective. Therefore, we activated Rg3 $CD8^{+}$ T cells in vitro with TB10.$4_{4–11}$ peptide, IL-2 and IL-12 and after 60–72 hours, transferred them into sublethally irradiated mice and infected them with *M. tuberculosis*. We compared the protective capacity of Rg3 $CD8^{+}$ T cells with ovalbumin-specific $CD8^{+}$ T cells (e.g., OT-I cells).

When a large number of activated cells (e.g., 10^{6}) were transferred, both Rg3 and OT-I cells transferred considerable protection (Fig 7A). The ability of large numbers of OT-I cells to transfer protection has not been directly investigated, but may be due to their highly activated state and could involve the production of IFNγ, has shown previously for ovalbumin-specific CD4+ T cells [29]. As the number of transferred cells was titrated down, protection mediated by OT-I cells diminished (Fig 7A). In contrast, Rg3 cells continued to mediate significant protection even when as few as 10,000 cells were transferred (Fig 7A). Transfer of Rg4 T cells also conferred protection against *M. tuberculosis* challenge (see S8A Data).

To demonstrate that protection required TCR dependent recognition of antigen, we transferred activated Rg3 cells (10^{6} cells/mouse) into sublethally irradiated WT or TAP1-/- recipient mice. Rg3 effector $CD8^{+}$ T cells were able to transfer protection to WT but not TAP1-/- recipients (Fig 7B). As in vitro activation bypasses the need for in vivo priming, these results show that protection mediated by Rg3 $CD8^{+}$ T cells requires recognition of antigens processed by the TAP-dependent class I MHC antigen-processing pathway.

We next determined which effector functions were required for protection. Rg3 mice were produced in an $IFN\gamma^{-/-}$ background and $IFN\gamma^{-/-}$ Rg3 $CD8^{+}$ T cells compared to WT Rg3 $CD8^{+}$

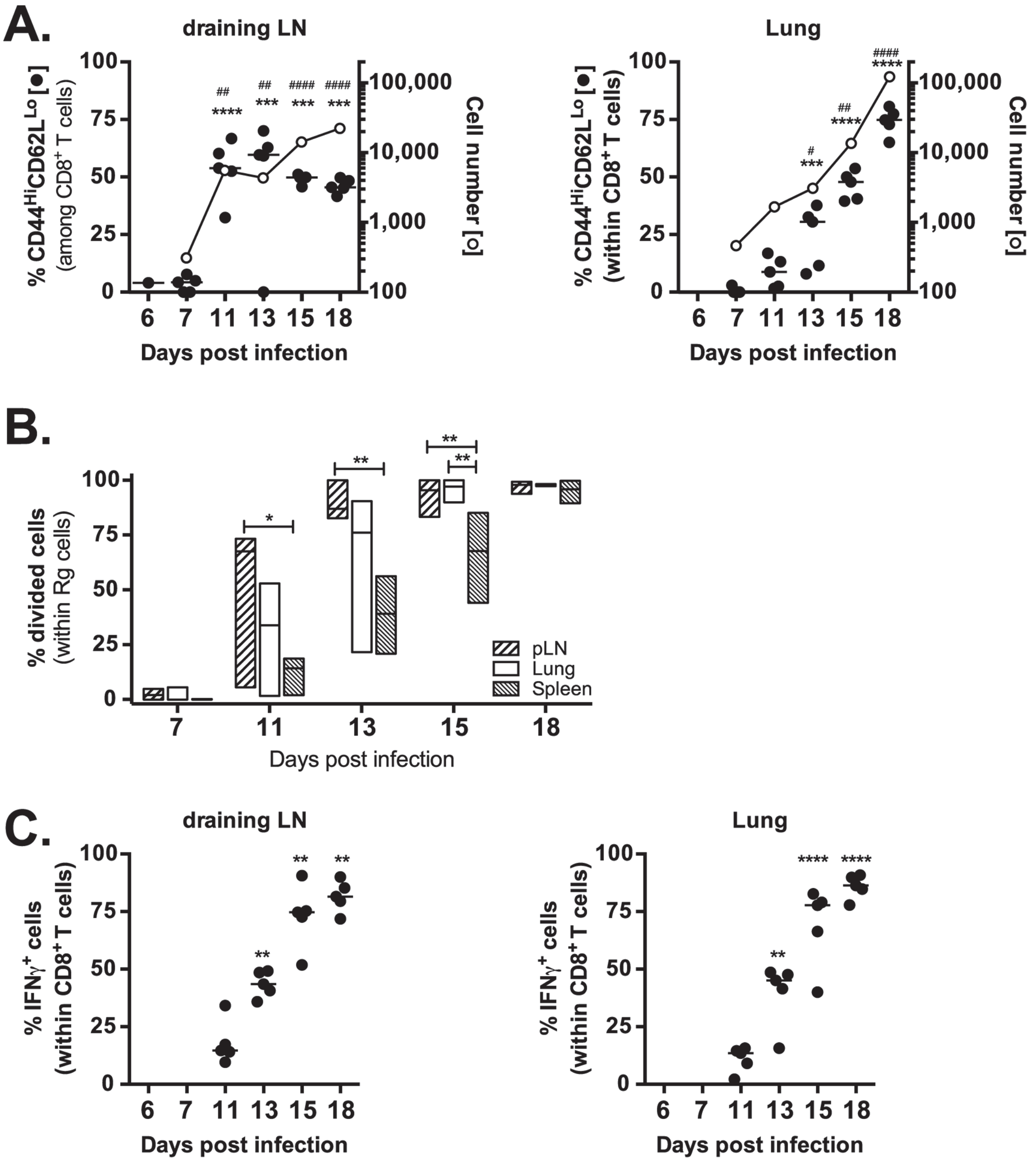

Fig 6. Retrogenic T cell priming and acquisition of effector functions. (**a**) Kinetic analysis of frequency (filled circles) and number (opened circles) of activated (CD44^{Hi}CD62L^{Lo}) Rg cells in the draining LN (left panel) and lung (right panel) following adoptive transfer into mice infected with *M*. *tuberculosis*. (**b**) Kinetic analysis of frequency of divided Rg cells in the draining LN, lung and spleen following adoptive transfer into mice infected with *M*. *tuberculosis*. (**c**) Kinetic analysis of frequency of IFNγ-producing Rg cells in the draining LN (left panel) and lung (right panel) following adoptive transfer into mice infected with *M*. *tuberculosis*. Data are representative from two (b) or three (a, c) independent experiments, each with 5 mice per group. (**a,c**) One way ANOVA with Dunnett's post test to compare differences over time (vs. day 7 [**a**] or d11 [**c**]) time points. P<0.05 indicated by asterisks (phenotype or IFNγ) or hash marks (cell numbers). (**b**) One way ANOVA with Tukey's post test to compare differences in proliferation between lung, LN and spleen; p<0.05 indicated by asterisks.

doi:10.1371/journal.ppat.1004849.g006

T cells for their ability to transfer protection. Under our experimental conditions, activated WT Rg3 CD8^{+} T cells (10^6 cells/mouse) transferred significantly more protection than OT-I or IFNγ$^{-/-}$ Rg CD8^{+} T cells (Fig 7C). These results show that transfer of protection by TB10.4-specific Rg CD8^{+} T cells requires IFNγ production (Fig 7C). Furthermore, naïve Rg3 CD8^{+} T cells (10^5 cells/mouse) prolonged the survival of *M*. *tuberculosis*-infected TCRα$^{-/-}$ mice in an

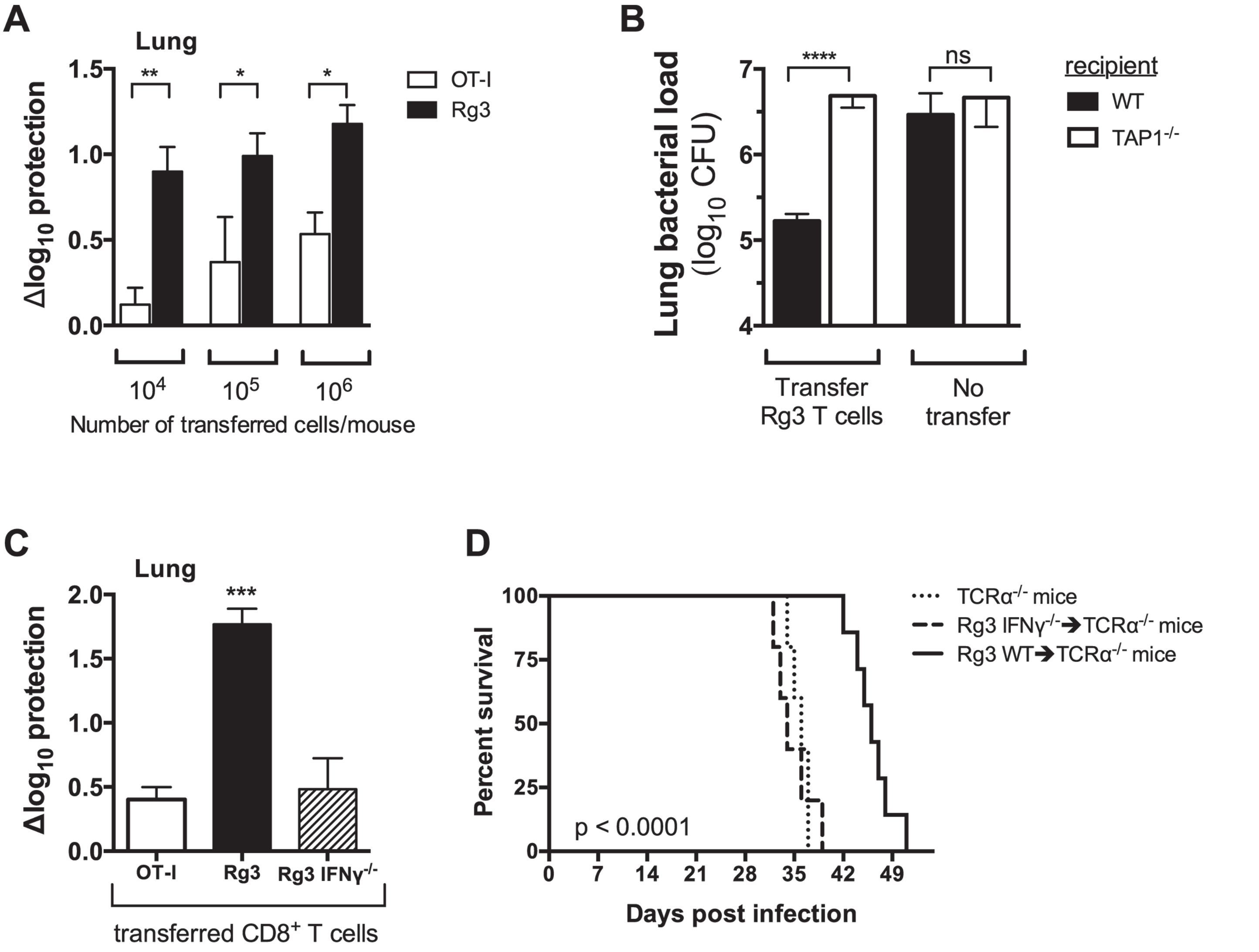

Fig 7. TAP1 and IFNγ are required for protection mediated by TB10-specific CD8+ T cells. (**a,b**) Bacterial burden in the lung 21 days after adoptive transfer of in vitro stimulated Rg (**a,b**) or OT-I (**a**) $CD8^+$ T cells into sub-lethally irradiated, *M*. *tuberculosis*-infected C57BL/6J (**a,b**) or TAP-1 deficient (**b**) recipients. (**c**) Bacterial burden in the lung 21 d after adoptive transfer of in vitro stimulated Rg, IFNγ-/- Rg, or OT-I $CD8^+$ T cells into sub-lethally irradiated, *M*. *tuberculosis*-infected C57BL/6J recipients. (**d**) Survival curves after adoptive transfer of naïve Rg or IFNγ-/- Rg $CD8^+$ T cells into *M*. *tuberculosis*-infected TCRα-/- recipients. Transfers used 10^6 cells/mouse in "b" and "c" or 10^5 cells/mouse in "d". Statistical significance calculated using the method of the Log-rank (Mantel-Cox) test. Data are representative from two independent experiments, each with at least 5 mice per group. Other statistical testing done by one-way ANOVA and ad hoc post tests. NS, not significant; *, p < 0.05.

doi:10.1371/journal.ppat.1004849.g007

IFNγ-dependent manner (Fig 7D). Collectively, these data show that TB10.4-specific $CD8^+$ T cells can control *M*. *tuberculosis* infection and IFNγ production after recognition of antigen presented in vivo is required for protection.

Differences in TCR avidity can determine clonotypic dominance during infection

Two of the cloned TCRs (Rg3 and Rg4) differed in their binding to K^b/TB10.4_{4-11} tetramers, revealing a difference in avidity (Fig 8A). No difference in the level of TCR level was detected (see S8B Data). Importantly, when transferred separately into intact mice, both Rg3 and Rg4

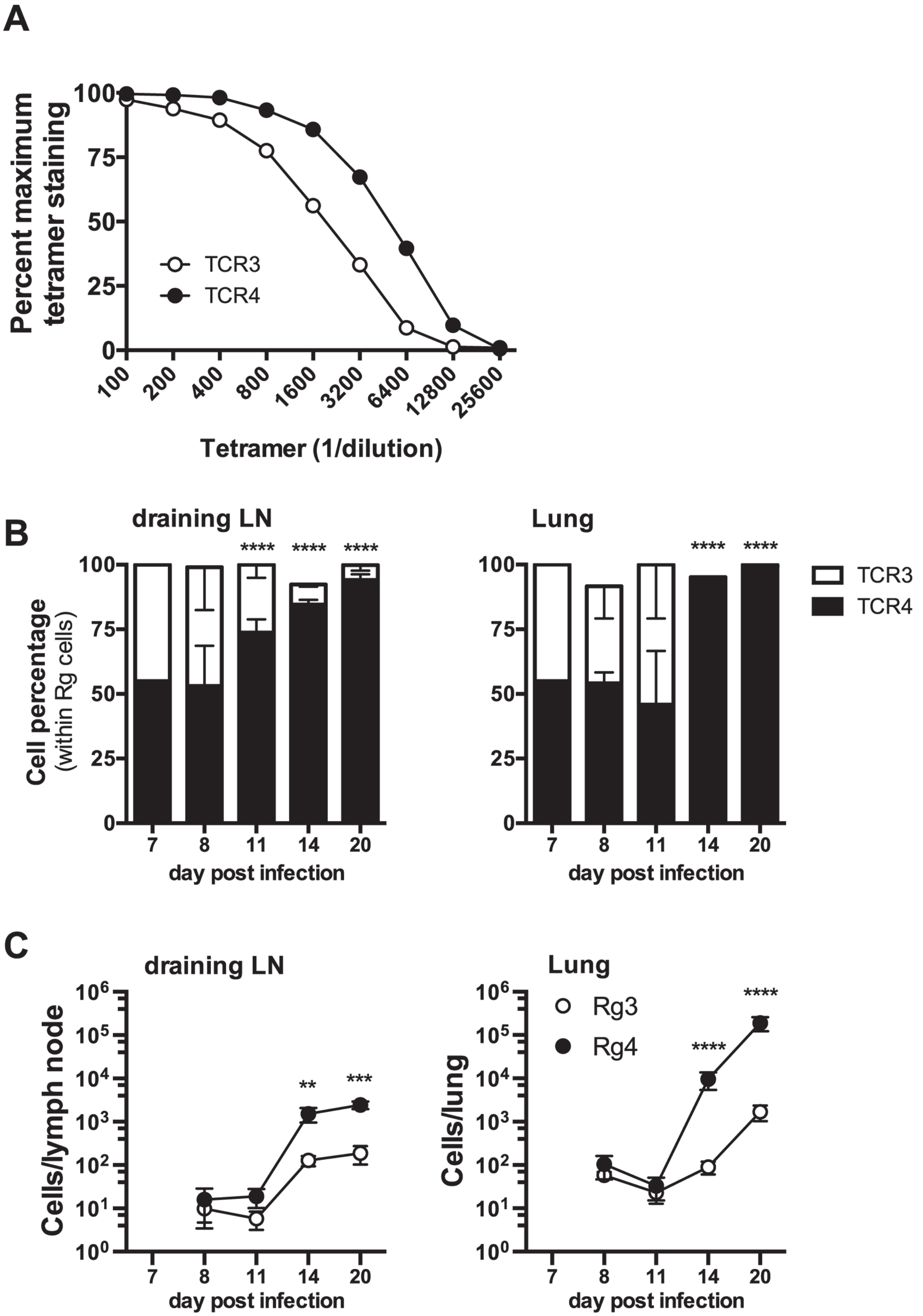

Fig 8. Differences in TCR affinity can lead to clonotypic dominance during infection. (**a**) Flow-cytometry analysis of affinity of Rg T cells from uninfected retrogenic mice expressing TCR3 (open circles) or TCR4 (filled circles), based on the frequency of tetramer staining of Rg cells across multiple tetramer concentrations. (**b,c**) Kinetic analysis of frequency (**b**) and number (**c**) of TCR3 (open symbols) or TCR4 (filled symbols) Rg cells in the draining LN (left panels) and lung (right panels) following adoptive co-transfer into mice infected with *M. tuberculosis*. Data are representative from two (b, c) or three (a) independent experiments, each with 5 mice per group. (**b, c**) Two way ANOVA with Holm-Sidak's multiple comparison test; *, $p < 0.05$).

doi:10.1371/journal.ppat.1004849.g008

were primed, underwent expansion and trafficked to the lung with similar kinetics, and mediated protection (Figs 6 and 7 and see S8 Data). To investigate whether TCR avidity could affect immunodominance, we co-transferred naïve Rg cells expressing either TCR3 or TCR4 at a 1:1 ratio into congenic recipients and analyzed their relative abundance following infection. Following co-transfer into *M*. *tuberculosis* infected mice, Rg3 and Rg4 T cells initially maintained a 1:1 ratio and importantly, both Rg3 and Rg4 T cells were primed in the LN and increased in number (Fig 8B and 8C). However, by day 11 post infection, Rg4 T cells began to outnumber Rg3 T cells, and by day 14, Rg4 T cells outnumbered Rg3 T cells by a ratio of 10:1 in the LN (Fig 8B and 8C). In the lung, there was no change in the ratio or cell count until day 14, at which point Rg4 $CD8^+$ T cells accounted for >90% of the Rg $CD8^+$ T cells (Fig 8B). While the absolute number of both Rg3 and Rg4 $CD8^+$ T cells increased in the lung by day 20, Rg4 $CD8^+$ T cells outnumber Rg3 $CD8^+$ T cells by 100:1 (Fig 8C). Although both Rg3 and Rg4 were able to expand and confer protection when transferred separately, Rg4 T cells dominated when in competition with Rg3 T cells. When co-transferred, both Rg3 and Rg4 $CD8^+$ T cells were primed in the LN and proliferated; however, small differences in their affinity led to the establishment of clonotypic dominance by Rg4 $CD8^+$ T cells during the T cell response to *M*. *tuberculosis* infection.

Discussion

We report that extreme TCR bias develops during the polyclonal $CD8^+$ T cell response to a single immunodominant epitope during tuberculosis in humans and in mice. In mice, preferential Vβ use by $TB10.4_{4-11}$-specific $CD8^+$ T cells is detected in the LN within 3 weeks of infection, indicating that bias develops soon after T cell priming. With time, TCR clonality becomes more extreme. Why do a few clonotypes dominate the $TB10.4_{4-11}$-specific $CD8^+$ T cells during *M*. *tuberculosis* infection?

TCR diversity arises by three principal mechanisms: 1) V, D, and J segments generate combinatorial diversity; 2) imprecise recombination and insertion of non-templated 'N' sequences at the VβD, DJβ and VαJα junctions; and 3) random assortment between TCRα and TCRβ chains [30,31]. By these mechanisms, people have the potential to generate $>10^{14}$ unique TCRαβ receptors [1,32]. Thymic selection constrains the repertoire by ensuring that T cells that are unable to recognize MHC and the T cells that recognize self-antigens with high affinity are deleted. As a result, people have ~10^{10} T cells and each clonotype is represented by 10–500 T cells; therefore, the number of unique TCRs in any individual (~2.5×10^7) is far fewer than the number of potential TCRs [2,3]. Although sharing of TCRs between people is improbable, such 'public' TCRs are detected and may have special significance [23].

If the number of antigen-specific T cells in the naïve repertoire is limiting, clonotypic dominance could arise by a "founder" effect in which few T cells are primed and expand, leading to T cell populations of restricted diversity. A founder effect is unlikely to explain the TCR bias among $TB10.4_{4-11}$-specific $CD8^+$ T cells because we detect ~900 naïve T cells in each C57BL/6 mouse, a precursor frequency that is among the highest recorded for antigen-specific $CD8^+$ T cells in the mouse [7]. $TB10.4_{4-11}$-tetramer$^+$ $CD8^+$ T cells from the LNs of infected mice use many Vβ families, which also argues against a founder effect. Another possibility is that the frequency of each clonotype in the naïve repertoire is skewed and results in TCR bias after infection. We observe considerable heterogeneity in the relative abundance of naïve precursors with the potential to recognize the $TB10.4_{4–11}$. However, skewing in the naïve repertoire cannot solely explain the selection that we observe for $TB10.4_{4-11}$-specific $CD8^+$ T cells following infection.

After infection, TB10.$4_{4\text{-}11}$-specific CD8$^+$ T cells express a limited number of CDR3β sequences. Given the high precursor frequency, we predicted *a priori* that the response would be dominated by private clonotypes, i.e., TCRs unique to each individual. Instead, common CDR3β motifs are generated by independent VDJ recombination events, within the same mouse and among different individuals. The most frequently used and shared TCRβs are under selective pressure, determined using a modification of Warren's method [33].

Developing retrogenic mice that over-produce CD8$^+$ T cells specific for TB10.$4_{4\text{–}11}$ permitted us to perform competition experiments with naïve TB10.$4_{4\text{-}11}$-specific CD8$^+$ T cells. A small difference in affinity significantly affects clonal representation and establishment of hierarchical dominance during infection. These data provide crucial experimental support for the idea of antigen-driven selection based on our TCR analysis. Although TCR affinity seems to be a major factor driving immunodominance, other factors can contribute. For example, the inflammatory environment and tissue-specific cues influence the fate of individual CD8+ T cells during infection [4], and these factors may contribute to the establishment of immunodominant T cell responses during tuberculosis.

Why is TB10.4 an immunodominant antigen? While there is a high precursor frequency, few of these T cell clones are significantly represented in the final immune response. The lack of a correlation between the precursor frequency and immunodominance in C57BL/6 and BALB/c mice [ref:10] indicates that a high precursor frequency is not a prerequisite for immunodominance during chronic infection. TB10.4 belongs to a larger family of ESAT6-related proteins that are secreted by specialized type VII secretion systems, and many of the secreted proteins are immunodominant in different animal species and humans [34–36]. While the abundance of TB10.4 during infection is unknown, paucity of this antigen during T cell priming or limited antigen presentation in the infected lung could drive clonotypic dominance by selecting for higher affinity T cells.

Whether certain mycobacterial antigens that elicit immunodominant T cell responses act as "decoys" to distract the immune response from responding to subdominant epitopes that might be more important targets of protective immunity was raised by Baena and Porcelli [37]. The finding that portions of the mycobacterial genome that encode T cell epitopes was more evolutionarily conserved has fueled this idea and raised the possibility that T cell immunity could benefit *M. tuberculosis*, possibly by creating an inflammatory environment that facilitates transmission [38]. Work done by the Andersen group finds that cryptic epitopes of ESAT6 are minor components of natural immune response to M. tuberculosis but specific vaccination strategies that elicit CD4+ T cells specific for the subdominant epitopes generate more durable protection against tuberculosis [39–41].

Could TCR diversity (or bias) be a surrogate for the quality or effectiveness of T cell immunity? Spectratyping of peripheral blood T cells from tuberculosis patients reveals TCR skewing compared to healthy controls [20,42,43]. Extreme TCR bias (e.g., clonality) was noted primarily in the setting of severe clinical disease, raising the possibility that TCR bias is associated with disease progression [19,20]. Arguing against this interpretation is the presence of highly skewed TCR repertoires in lung granulomas from patients with latent tuberculosis [17]. In our cohort of patients, we also see the emergence of clonal T cell expansions in the lungs of patients with active disease, compared to the frequency of T cells in the peripheral blood of normal donors. Importantly, clonal expansions can be detected among peripheral blood CD4$^+$ and CD8$^+$ T cell in uninfected healthy individuals, with memory T cells being 50-fold less diverse than naive T cells [33]. While these data indicate that T cell expansions can occur independently of active infection, clonotypes expressing the CDR3β motif "DREN" were 1000-fold enriched in Mtb granulomas compared to their average frequency in the peripheral blood of normal donors. Thus, these expansions are several orders of magnitude greater than the expansions

reported in uninfected individuals [33]. This may indicate that the "DREN" expansions are driven by Mtb infection. Furthermore, based on our murine data, we infer that T cell selection, possibly driven by affinity, is occurring. We are confident that we captured the majority of lung TB10.4-specific CD8+ T cells present in each individual mouse. In contrast, we observed considerable heterogeneity in the TCR repertoire obtained from distinct granulomas in each human subject. While this heterogeneity is not surprising based on the work on Flynn and Barry [44,45], it is important to recognize that TCR bias at the level of the granuloma may be driven by heterogeneity in bacteria and bacterial antigens, as well as the persistent immune response. Therefore, the links between TCR bias, functional capacity of T cells and protection during tuberculosis are still unclear, and longitudinal studies coupled with the functional study of antigen-specific T cells are needed to define what constitutes a protective T cell response against tuberculosis in both people and in experimental animal models, both in terms of TCR diversity and T cell function [46].

While antigen choice is a key part of vaccine development, predicting which immunogenic epitopes elicit memory responses that generate protective immunity during infection has not been straightforward. Some pathogens mutate to escape T cell surveillance; other pathogens avoid immune detection by sequestering their antigens. $CD4^+$ and $CD8^+$ T cells specific for TB10.4 are elicited following clinical tuberculosis infection. Based on the ability of TB10.4-specific $CD4^+$ T cells to mediate protection in animal models, the TB10.4 antigen has been incorporated into subunit vaccines. Our data is the first to show that TB10.4-specific $CD8^+$ T cells transfer protection and that protection requires antigen presentation by TAP1-dependent pathways. This implies that the TCR-mediated recognition of infected cells is a prerequisite for the antimicrobial activity of CD8+ T cells. Furthermore, IFNγ is a key mediator of bacterial control.

Why then did a vaccine designed to elicit TB10.4-specific $CD8^+$ T cells fail to protect mice against *M*. *tuberculosis* [47]? We chose to measure protection in immunocompromised (e.g., sublethally irradiated) mice, as it is difficult to demonstrate CD8+ T cell mediated protection in mice with an intact CD4+ T cell compartment. Another factor that may impair the ability of TB10.4-specific CD8+ T cells to protect mice with an intact immune system is inefficient presentation of IMYNYPAM. Lindenstrom et al show that vaccination with native TB10.4 protein does not elicit TB10.4-specific CD8+ T cells because the amino acid sequence surrounding the epitope (e.g., IMYNYPAM**L**) is inefficiently processed and presented. In contrast, a homologous protein (TB10.3, EsxR) contains a homologous sequence (e.g., IMYNYPAM**M**), which is more efficiently processed and presented. Less TB10.3 is produced by the bacterium than TB10.4, and because of this, Lindenstrom speculates that IMYNYPAM is not presented efficiently by infected macrophages. Our own data showing protection (Fig 7) used cells activated in vitro prior to adoptive transfer, bypassing the need for priming. TAP1-dependent protection mediated by these TB10.4-specific CD8+ T cells implies that infected cells in the lung present IMYNYPAM. However, the source of the antigen (e.g., TB10.3 vs. TB10.4) is uncertain. If IMYNYPAM-specific CD8+ T cells recognize the small amount of TB10.3 expressed by infected macrophages, then selecting CD8+ T cells with a high affinity for IMYNYPAM will be even more important for host resistance.

Our study took advantage of the well-characterized $CD8^+$ T cell response to Mtb in mice, where responses to an epitope of TB10.4 elicit an immunodominant T cell response. This allowed us to track and purify antigen-specific $CD8^+$ T cells during infection. We find that the TB10.4-specific $CD8^+$ T cell response is characterized by extreme clonality despite originating from a high-frequency naïve precursor pool. We were able to show that structural features of the CDR3β region were important for epitope recognition, most likely because of clonal competition and affinity selection. Similarly, we found that human $CD8^+$ T cells also undergo

selection and clonal expansion. Although the clonal expansions we detected in humans were not as dramatic as in the mouse model, we believe that this is partially because we were unable to purify human antigen-specific $CD8^+$ T cells, due to the lack of appropriate reagents. The ability to use tetramer-sorted cells allows one to analyze T cells that are all specific for a single epitope, which adds considerable power to the TCR analysis. Therefore, it is uncertain whether the extreme immunodominance we observe in the murine system will be found in humans, and this is the subject of active investigation. For example, greater T cell diversity is theoretically expected in humans, due to the contributions of HLA variability, but also empirically, as demonstrated by the large-scale studies to date that have generally found a more diverse T cell response in infected individuals [48,49]. However, this may also be due to technical barriers; while antigen-specific CD8+ T cell expansions are found during tuberculosis, they often appear to be unique to individuals and their private repertoires, which further complicates TCR analysis of antigen-specific T cells [48–50]. If this holds true, it could be difficult to fully exploit TCR analysis as a biomarker for following disease progression or treatment efficacy, although if expanded T cell clones from the lung are correlated with those that are present (and can be detected) in peripheral blood, algorithms might be developed that are independent of antigen-specificity.

Finally, based on the propensity of Mtb to drive clonal selection of CD8+ T cells, we infer that there is a paucity of antigen presentation in the infected lung. Such a state may arise because of inefficient cross-presentation of antigens by the class I MHC-processing pathway and could explain why CD8+ T cells have not proven to be as protective as CD4+ T cells. Similarly, if low levels of antigen presentation rapidly select for high affinity $CD8^+$ T cells during infection, an effective vaccine will need to elicit similarly high affinity T cells, rather than large numbers of diverse T cells, if they are to be effective in controlling bacterial replication.

Materials and Methods

Ethics statement

The University of KwaZulu Natal (UKZN) Biomedical Research Ethics Committee (BREC) approved the study protocol, its associated informed consent documents and data collection tools. Written informed consent was obtained for all research subjects.

All animal experiments were performed in accordance with National and European Commission guidelines for the care and handling of laboratory animals. The studies were approved by the Institutional Animal Care and Use Committee at the Dana Farber Cancer Institute and the University of Massachusetts Medical School (Animal Welfare Assurance no. A3023-01 [DFCI] or A3306-01 [UMMS]), under Public Health Service assurance of Office of Laboratory Animal Welfare guidelines).

Human granuloma T cells

Lung tissue of approximately 3 cm^3 was isolated from different areas of resected lungs, corresponding to the most diseased (A), intermediate (B), and healthiest tissue (C), typically corresponding the upper (A), lower (B) and middle lobe (C) (Table 1). The operating surgeon classified the tissue based on their experience and the pre-operative radiological data. Each sample was washed in multiple changes of HBSS and then diced into approximately 1 mm^3 pieces, which were strained, re-suspended in 7mls of pre-warmed digestion media (R10 (RPMI supplemented with 10% FCS, 2 mM L-glutamate, 100 U/ml Penstrep), containing 0.5 mg/ml collagenase D (Roche) and 40 U/ml DNaseI (Roche), and transferred to GentleMACS C-tubes (Miltenyi) for mechanical digestion per manufacturers instructions. The resultant suspension was incubated for 60 min at 37 °C, subjected an additional mechanical digestion step. The

resulting suspension was strained through a 70 μm cell strainer, washed twice in HBSS, stained and $CD8^+$ T-cells sorted using the FACS ARIA system, gating on the live (nearIR, Invitrogen) singlet, $CD45^+$, $CD3^+$ and $CD4^-$ population. Cells were sorted directly into RLT buffer, and genomic DNA extracted using the DNeasy Minikit (Qiagen) as per manufactures instructions.

Mice

C57BL/6 (WT), CD45.1 (B6.SJL-$Ptprc^a Pepc^b$/BoyJ), CD90.1 (B6.PL-$Thy1^a$/CyJ), OT-I (C57BL/6-Tg(TcrαTcrβ)1100Mjb/J), TCRα KO (B6.129S2-$Tcra^{tm1Mom}$/J), IFNγ KO (B6.129S7-$Ifng^{tm1Ts}$/J) and TAP KO (B6.129S2-$Tap1^{tm1Arp}$/J) mice were purchased from Jackson Laboratories (Bar Harbor, ME). Va2var mice [24] were bred at Jackson Laboratories (Bar Harbor, ME). Mice were 6 to 10 weeks old at the start of all experiments. Mice infected with *M. tuberculosis* were housed in a biosafety level 3 facility under specific pathogen-free conditions at DFCI or at UMMS.

Experimental infection and bacterial quantification

M. tuberculosis (Erdman strain) infection was performed via the aerosol route, and mice received 50–200 CFU/mouse. A bacterial aliquot was thawed, sonicated twice for 10 s in a cup horn sonicator, and then diluted in 0.9% NaCl–0.02% Tween 80. A 15 ml suspension of *M. tuberculosis* was loaded into a nebulizer (MiniHEART nebulizer; Vortran Medical Technologies) and mice were infected using a nose-only exposure unit (Intox Products). Alternatively, the bacterial aliquot was diluted in a final volume of 5ml, and mice were infected using a Glas-Col aerosol-generation device. At different times post-infection, mice were euthanized by carbon dioxide inhalation, organs were aseptically removed, individually homogenized and viable bacteria were enumerated by plating 10-fold serial dilutions of organ homogenates onto 7H11 agar plates. Plates were incubated at 37°C and *M. tuberculosis* colonies were counted after 21 d.

FACS analysis and cell sorting

Cell suspensions from lung, spleen and LNs were prepared by gentle disruption of the organs through a 70 μm nylon strainer (Fisher) or using the GentleMacs apparatus (Miltenyi Biotec, Germany) according to the manufacturer instructions. For lung preparations, tissue was digested for 30–60 min at 37 °C in 1 mg/mL collagenase (Sigma) prior to straining. Erythrocytes were lysed using a hemolytic solution (155 mM NH_4Cl, 10 mM $KHCO_3$, 0.1 mM sodium EDTA pH 7.2) and, after washing, cells were resuspended in supplemented RPMI (10% heat inactivated FCS, 10 mM HEPES, 1 mM sodium pyruvate, 2 mM L-glutamine, 50 mg/ml streptomycin and 50 U/ml penicillin, all from Invitrogen) or MACS buffer (Miltenyi Biotec, Germany). Cells were enumerated in 4% trypan blue on a hemocytometer or using a MACSQuant flow cytometer (Miltenyi Biotec, Germany). Surface staining was performed with antibodies specific for mouse CD3 (clone 17A2), CD3ε (clone 145-2C11) CD4 (clone GK1.5), CD8 (clone 53–6.7), CD19 (clone 6D5), CD44 (clone IM7), CD62L (clone MEL-14), CD45.1 (clone A20), CD45.2 (clone 104), CD90.1 (clone OX-7), CD90.2 (clone 53–2.1), Vα2 (clone B20.1), Vβ4 (clone KT4), Vβ5 (clone MR9-4), Vβ7 (clone TR310), Vβ10 (clone B21.5), Vβ11 (clone RR3-15) (from Biolegend or BD Pharmingen, CA, USA). The specificity of the anti-Vα and-Vβ mAbs are shown in Table 2. The tetramers $TB10.4_{4-11}$-loaded H-2 K^b were obtained from the National Institutes of Health Tetramer Core Facility (Emory University Vaccine Center, Atlanta, GA, USA). All stainings were performed for 20 min on ice and, unless stated, cells were fixed before acquisition with 2% formaldehyde in PBS for 30–60 min. Cell analysis was performed on a FACS Canto (Becton Dickinson, NJ, USA) or on a MACSQuant flow cytometer (Miltenyi Biotec, Germany). Data were analyzed using FlowJo Software (Tree Star, OR, USA).

For cell sorting, stained and non-PFA fixed cells were suspended in MACS buffer (Miltenyi Biotec, Germany) and deposited in collection tubes using a BD Canto flow cytometer (Becton Dickinson, NJ, USA). For both FACS analysis and cell sorting, single-lymphocyte events were gated by forward scatter versus height and side scatter for size and granularity.

Next generation sequencing

For TCRβ high-throughput sequencing, genomic DNA was purified from sorted cell populations and sequenced by Adaptive Biotechnologies Corp. (Seattle, WA) using the ImmunoSEQ assay (http://www.immunoseq.com) as previously described[51]. Data were analyzed using the ImmunoSEQ analyser toolset. Clonality is the entropy of the TCRB frequency distribution and is calculated as 1-(entropy/log_2[# unique TCRs]). Here entropy, a measure of diversity within a complex data set, is also known as the Shannon-Wiener index, Shannon's diversity index or Shannon's entropy [52,53]. Thus "0" represents a diverse repertoire and "1" is a completely clonal repertoire.

Enumeration of naïve Ag-specific T cells

Analysis of the precursor frequency of naïve T cells was performed as previously described [54]. Briefly, the spleen and axillary, mesenteric, cervical, inguinal, popliteal, and salivary LN were harvested from individual mice, dispersed, and filtered through a 70-mm mesh and enumerated for total and $CD8^+$ T cell composition. The cell suspension was then costained with identical PE- and APC-conjugated tetramers and then purified via anti- PE magnetic bead selection (Miltenyi Biotec, Germany). Positive and negative fractions were then surface stained with anti-MHC II, CD11b, CD19, and CD4 as a "dump" channel, and anti-CD8α, CD3 and CD44. Flow cytometry counting beads were added immediately before samples were collected by the cytometer to determine the fraction of tetramer+ events collected and used to determine the total number and frequency of tetramer+ cells in each animal. For analysis purposes, naïve cells were cells that were present in the bound fraction, costained with PE- and APC-conjugated tetramers, and did not express CD44.

Single cell sorting and single cell PCR

Live lymphocytes were sorted as $TCR\beta^+CD8^+Tet^+CD19^-CD4^-CD11b^-CD11c^-$ as individual cells into wells of 96-well PCR plates containing 10 μl of reverse transcriptase buffer (50 mM Tris-HCl, 75 mM KCl, and 3 mM MgCl2), 2% Triton X-100, 500 μM dNTP with 1 μg BSA, 50 ng of oligo(dT) (12–18), 500 μM dTT, 50 μM of TCRα-specific primer, 50 μM of TCRβ-specific primer, 8 U of RNaseOUT, and 30 U of Moloney murine leukemia virus reverse transcriptase (Invitrogen Life Technologies). The plates were incubated for 90 min at 37°C, then heat inactivated for 20 min at 80°C. 2 μl of the cDNA were used for each of the nested PCRs for TCRα or TCRβ (see S9 Data for list of primers). The first round of each nested PCR amplification was performed by combining 2 μl of cDNA with 9 μl of *Taq* buffer (50 mM KCl, 10 mM Tris-HCl (pH 8.3), and 2.5 mM MgCl2), 500 μM dNTP, 0.3 U of *Taq* polymerase, 50 μM of $TRAC_{ext}$- or $TRBC_{ext}$-specific primer for the constant region and an oligonucleotide mixture of 23 $TRAV_{ext}$ or 19 $TRBV_{ext}$ primers (each 50 μM final concentration). For the second round of the nested reaction, 2 μL of the first reaction were combined with 18 μL of *Taq* buffer (50 mM KCl, 10 mM Tris-HCl (pH 8.3), and 2.5 mM MgCl2), 500 μM dNTP, 0.6 U of *Taq* polymerase, 50 μM of $TRAC_{int}$- or $TRBC_{int}$-specific primer for the constant region and an oligonucleotide mixture of 23 $TRAV_{int}$ or 19 $TRBV_{int}$ primers (each 50 μM final concentration). The PCR conditions for the first round of PCR were 94°C for 3 min followed by 35 cycles of 94°C for 20 s, 52°C for 45 s, and 72°C for 60 s, with a final extension at 72°C for 7 min. For the second round

of PCR conditions were the same, but only for 26 PCR cycles. Contamination was monitored for all steps (sorting, reverse transcriptase, and PCR), by leaving 16 control wells empty per 96-well PCR plate sorted.

For TCR product sequencing, a total of 12 μl of the products from the second round of the nested PCR amplification was combined with 1.5 μl of 10X shrimp alkaline phosphatase reaction buffer (200 mM Tris-HCl (pH 8.0) and 100 mM MgCl2), 1 U of shrimp alkaline phosphatase (Amersham Biosciences), and 1 U of exonuclease I (New England Biolabs), and water to total 15 μl. The reaction was then heated to 37°C 30 min, 80°C 10 min, and cooled to 4°C, and the product was subjected to automated sequencing (Dana-Farber/Harvard Cancer Center High-Throughput Sequencing Core). The sequences of the four TCRs cloned are shown in S10 Data.

Generation of retrogenic mice

TCR retroviral constructs were generated as 2A-linked single open reading frames using PCR and cloned into a murine stem cell virus-based retroviral vector with a GFP marker as previously described [55,56]. Details of cloning strategies and primer sequences are available upon request (samuel.behar@umassmed.edu). Retroviral-mediated stem cell gene transfer was performed as previously described [55,56].

Intracellular cytokine staining

5×10^5 cells were plated in each well of a round bottom 96-well plate and incubated in the presence of TB10.4_{4-11} peptide (10 μM; New England Peptide). Incubation in the presence of αCD3/αCD28 (1 μg/mL; BioLegend) or in the absence of stimuli were used as positive and negative controls, respectively. Cells were incubated for 1 h at 37° C, at which point Golgi Stop solution (BD Pharmingen, CA, USA) was added to each well for the remaining 4 h. Cells were collected after the 5 h stimulation and then surface stained with the antibodies described above, followed by intracellular staining for IFNγ (clone) using BD Permwash Kit (BD Pharmingen, CA, USA) as per manufacturer's instructions.

In vitro CTL assay

In vitro cytotoxicity was determined using peptide- coated EL4 target cells differentially labeled with the cell proliferation dye efluor 450 (eBiosciences) as previously described [21]. Briefly, target cells were pulsed with 10 μM of TB10.4 peptide at 37°C for 1 h in complete medium or left unpulsed (as controls), followed by extensive washing. Target cell populations were then labeled with either 10 μM or 100 nM e450 dye in PBS for 20 min at room temperature, followed by extensive washing. Labeled populations were mixed at an equal cell ratio with effector retrogenic cells at 1:1:1 ratio in round bottom 96-well plates (100,000 cells/population, in triplicate). Plates were incubated for 4–12 h at 37°C, in the dark. After incubation, the cells were analyzed by flow cytometry, and the ratios of recovered GFP (retrogenic, effector) and e450-labeled target EL4 populations were determined.

Adoptive T cell transfer for priming and competition

Single cell suspensions of pools of spleens and LNs from naive retrogenic mice (6 to 10 wks post reconstitution) were prepared. $CD8^+$ T cells were purified from each suspension using the $CD8^+$ T cell isolation kit and magnetic separation (Miltenyi Biotec, Germany). After purification, cells were counted and transferred via the tail vein into congenically marked recipients (CD90.1), which had been infected 6–7 d earlier with virulent *M. tuberculosis* (Erdman) via the

aerosol route. For priming experiments, 10^4 to 10^5 cells were transferred into each recipient. For competition experiments, cells were mixed at a 1:1 ratio (confirmed by FACS analysis prior to injection) and then transferred into each recipient.

Measurement of cell proliferation

For analysis of cell proliferation of retrogenic cells after adoptive transfer, bead-purified naïve Rg cells (see above) were labeled with 10 μM of the cell proliferation dye efluor 450 (eBiosciences) in PBS for 20 min at room temperature, followed by extensive washing.

In vitro activation of T cells

Single cell suspensions of pools of spleens and LNs from naive retrogenic or OT-I mice were prepared. $CD8^+$ T cells were purified from each suspension using the $CD8^+$ T cell isolation kit and magnetic separation (Miltenyi Biotec, Germany). After purification, cells were counted and mixed at a 1:1 ratio with peptide coated APCs in media containing 100U/mL of IL-2 and 10U/mL of IL-12. APCs used were red blood cell lysed splenocytes from naïve C57Bl6/j mice, pulsed with 10 μM of TB10.4 or SIINFEKL peptide at 37°C for 1 h in complete medium, followed by irradiation with 3200 Rads from a cesium-137 source and extensive washing. $2x10^6$ cells were plated, 1 mL into each well of a 24 well plate. After 24 h, cells were fed with 1 mL of fresh media, containing 100U/mL of IL-2 and 10U/mL of IL-12. 48 h after the initial stimulation, cells were fed by removing 1 mL of culture media and addition of 1 mL of fresh media, containing 100U/mL of IL-2. 60–72 h after the initial stimulation, cells were extensively washed with complete media, and used for adoptive transfer experiments.

Adoptive T cell transfer for protection and survival

An adoptive transfer model was used to analyze the ability of T cells to mediate protection against pulmonary *M*. *tuberculosis* infection as previously described [21]. Briefly, C57BL/6 or TAP-ko mice were sublethally irradiated with 600 rad using a cesium-137 source. The next day, 10^4 to 10^6 bead-purified activated Rg T cells (or OT-1 cells, as controls) were transferred via the tail vein. Mice were infected with virulent *M*. *tuberculosis* (Erdman) via the aerosol route within 24 h of the adoptive T cell transfer. Three weeks after infection, the bacterial burden in the lung and spleen was determined. For survival experiments, naïve T cells (10^5 per mouse) were transferred into TCRα KO mice via the tail vein; following transfer, mice were infected with virulent *M*. *tuberculosis* (Erdman) via the aerosol route.

Statistical analysis

Population medians were used to compare TCR frequencies. Other data are represented as mean + SEM. For data with a verified for Gaussian distribution, a t-test was performed to compare two groups; otherwise, a Mann-Whitney *U* test was used. To compare more than 2 groups, one-way ANOVA, followed by Bonferroni post-hoc test was performed. Differences with a $p<0.05$ were considered significant and represented by *.

Supporting Information

S1 Data. Deep Sequencing of human TCRs from TB granulomas. Summary of deep sequencing data of human TCRβs from lung granulomas and lymph node resected from subjects with tuberculosis.
(PDF)

S2 Data. Clonality and clonality distribution. A. The Vβ gene distribution is shown for two samples: (top) CD8+ T cells from a human lung granulomas; and (B) PBMC from a normal donor. One can see how differences in the Vβ gene use affects the calculation of clonality can be visualized. Numbers do not add to 100% because only productive recombination events are plotted. Width axis: V gene; Depth Axis: CDR3 length; Height axis: frequency. **B.** The Vβ gene distribution is shown for four samples from a single subject (#23): lung sample A, B, and C; and LN. Although there are considerable differences between the samples, particularly with respect to TCR diversity (see Fig 1), the most abundant TCRs are shared between the different lung lesions and to a lesser extent, the LN sample. Again, one can visualize how the Vβ gene use affects clonality. Width axis: V gene; Depth Axis: CDR3 length; Height axis: frequency.
(PDF)

S3 Data. TCRβ frequencies in human lung granulomas. The TCRβ frequencies, derived for the frequency of unique DNA sequences, in each sample analyzed by deep sequencing. The bar is the median and the error bars denote the interquartile range. For comparison, the TCRβ frequencies in PBMC from three normal donors are shown. Only productive recombination events are plotted.
(PDF)

S4 Data. DREN motif in human TCRs. The frequency of the "DREN" motif in the CDR3 region of TCRβ chains sequenced from human granulomas or PBMC samples from normal individuals. Multiple unique sequences were detected in these samples. The frequency of "DREN" motifs was elevated in lung granuloma compared to peripheral blood. Statistical testing after $\log_{10}$ transformation showed that these differences were significant ($P<0.001$) for all pairwise combinations by one-way ANOVA with Tukey's multiple comparison test. These data show that TCRβ chains with the "DREN" CDR3 motif are increased in frequency among $CD8^{+}$ T cells in tuberculous lung granulomas compared to peripheral blood T cells from healthy controls.
(PDF)

S5 Data. Infection of Vα2var mice with Mtb. A. Schematic of the Vα2var locus of Vα2var mice. **B**. The $TB10.4_{4\text{-}11}$-specific $CD8^{+}$ T cell response following aerosol infection of WT vs. Vα2var mice. **C**. Survival of C57BL/6, Vα2var, or TCRα knockout mice after low dose aerosolized Mtb.
(PDF)

S6 Data. Frequency of arginine and aspartic acid in naïve cells. To determine whether the enrichment of "R" or "D" was significant, we compared their occurrence in the normal TCRβ repertoire. We queried the splenic TCRβ repertoire from three C57BL/6 mice representing over 1.1 million reads and ~53,000 unique sequences each. The average frequency of "R", "D", or "RD" at CDR3β position 6, 7, or 6–7, was 10.1%, 7.5%, and 2.5% respectively, indicating "R," "D," and "RD," were significantly enriched in the clonally expanded $TB10.4_{4\text{-}11}$-specific $CD8^{+}$ T cells. Testing using the Chi-square with Yates' correction shows that the frequency of R, D, or RD in the clonally expanded sequences is significantly increased in the four cloned sequences compared to the normal splenic repertoire of B6 mice ($P<0.0001$).
(PDF)

S7 Data. Effector function of Rg3 $CD8^{+}$ T cells. Rg3 CD8+ T cells were transferred to Mtb infected mice as described in the methods. Three to six weeks after aerosolized Mtb infection, lung cells were stimulated with the TB10.4 epitope. Intracellular staining for IFNg, IL-2, TNF, and granzyme were performed. The results shown are gate on Rg3 CD8+ T cells identified by

their co-expression of GFP and Va2
(PDF)

S8 Data. Rg4 CD8+ T cells mediate protection and are primed in the lungs of Mtb infected mice. A. Protection mediated by 10^6, 10^5 or 10^4 in vitro activated Rg4 (e.g., TCR4) $CD8^+$ T cells. T cells were transferred by the IV route into sublethally irradiated mice and challenged with Mtb. CFU were determined ~ 4 wks after infection. Protection (Δlog10) is the lung CFU in mice that did not receive Rg T cells minus the lung CFU in mice that did receive Rg T cells. Two independent experiments are shown each with 5 mice. One way ANOVA with Tukey's post test to compare differences in CFU. *, $p<0.05$; ***, $p<0.001$. **B**. The level of TCRα staining on Rg3 vs. Rg4 CD8+ T cells from the lungs of infected mice. T test showed the difference was not significant. **C**. Priming of naïve Rg4 and Rg3 CD8+ T cells in the pulmonary LN of Mtb infected mice occurs with similar kinetics. Rg3 and Rg4 CD8+ T cells were co-transferred or Rg3 CD8+ T cells were transferred alone. Priming is indicated by acquisition of an activated ($CD44^+CD62L^{lo}$ phenotype.
(PDF)

S9 Data. Primers used for single cell PCR analysis of TCRs used by murine CD8+ T cells. List of the TCRα and TCRβ primers used for nested PCR for the single cell analysis of TCRs used by TB10-specific CD8+ T cells from the lungs of Mtb-infected mice.
(PDF)

S10 Data. Retrogenic TCR sequences. TCR sequences and 2A peptide cloned into retroviral vectors. Sequences of four TCR sequences as assembled and cloned into the retroviral vectors. The first sequence is color-coded. VαJα (blue); Cα (purple); P2A (grey); VβDJβ (yellow); Cβ (green).
(PDF)

Acknowledgments

The authors would like to thank D. Vignali for kindly providing the reagents for retrogenic mice. We would like to thank C. Benoist, D. Cipoletta, D. Kolodin and J. Shu for their critical input and assistance with single cell sorting and generation of retrogenic mice. We would like to thank the flow cytometry cores at the Joslin Diabetes Center, at the Beth Israel Deaconess Medical Centre and the UMass Medical School for technical assistance with flow cytometry and sorting. We thank D. Hamm at Adaptive Technologies for the TCR sequence data on naïve mice and helpful discussions on its analysis. We thank J. Yang and J.F. Neves for critical reading of the manuscript.

Author Contributions

Conceived and designed the experiments: CNA MGB SMC ACR CJM MCN SMB. Performed the experiments: CNA MGB SMC ACR CJM DD KS HNK RM DR. Analyzed the data: CNA AL SMB. Wrote the paper: CNA MGB SMC AR CJM DD KS HNK RM DR AL MCN SMB.

References

1. Davis MM, Bjorkman PJ (1988) T-cell antigen receptor genes and T-cell recognition [published erratum appears in Nature 1988 Oct 20;335(6192):744]. Nature 334: 395–402. PMID: 3043226
2. Venturi V, Price DA, Douek DC, Davenport MP (2008) The molecular basis for public T-cell responses? Nature Reviews Immunology 8: 231–238. doi: 10.1038/nri2260 PMID: 18301425

3. Jenkins MK, Chu HH, McLachlan JB, Moon JJ (2010) On the composition of the preimmune repertoire of T cells specific for Peptide-major histocompatibility complex ligands. Annual Review of Immunology 28: 275–294. doi: 10.1146/annurev-immunol-030409-101253 PMID: 20307209
4. Plumlee CR, Sheridan BS, Cicek BB, Lefrancois L (2013) Environmental cues dictate the fate of individual CD8+ T cells responding to infection. Immunity 39: 347–356. doi: 10.1016/j.immuni.2013.07.014 PMID: 23932571
5. Obar JJ, Khanna KM, Lefrancois L (2008) Endogenous naive CD8+ T cell precursor frequency regulates primary and memory responses to infection. Immunity 28: 859–869. doi: 10.1016/j.immuni.2008.04.010 PMID: 18499487
6. Kotturi MF, Scott I, Wolfe T, Peters B, Sidney J, et al. (2008) Naive precursor frequencies and MHC binding rather than the degree of epitope diversity shape CD8+ T cell immunodominance. J Immunol 181: 2124–2133. PMID: 18641351
7. Jenkins MK, Moon JJ (2012) The role of naive T cell precursor frequency and recruitment in dictating immune response magnitude. J Immunol 188: 4135–4140. doi: 10.4049/jimmunol.1102661 PMID: 22517866
8. Blythe MJ, Zhang Q, Vaughan K, de CR Jr., Salimi N, et al. (2007) An analysis of the epitope knowledge related to Mycobacteria. ImmunomeRes 3: 10. PMID: 18081934
9. Majlessi L, Rojas MJ, Brodin P, Leclerc C (2003) CD8+-T-cell responses of Mycobacterium-infected mice to a newly identified major histocompatibility complex class I-restricted epitope shared by proteins of the ESAT-6 family. Infection and Immunity 71: 7173–7177. PMID: 14638811
10. Woodworth JS, Shin D, Volman M, Nunes-Alves C, Fortune SM, et al. (2011) Mycobacterium tuberculosis directs immunofocusing of CD8+ T cell responses despite vaccination. J Immunol 186: 1627–1637. doi: 10.4049/jimmunol.1002911 PMID: 21178003
11. Hoang TTKT, Nansen A, Roy S, Billeskov R, Aagaard C, et al. (2009) Distinct differences in the expansion and phenotype of TB10.4 specific CD8 and CD4 T cells after infection with Mycobacterium tuberculosis. PLoS ONE 4: e5928. doi: 10.1371/journal.pone.0005928 PMID: 19529765
12. Axelsson-Robertson R, Weichold F, Sizemore D, Wulf M, Skeiky YAW, et al. (2010) Extensive major histocompatibility complex class I binding promiscuity for Mycobacterium tuberculosis TB10.4 peptides and immune dominance of human leucocyte antigen (HLA)-B*0702 and HLA-B*0801 alleles in TB10.4 CD8 T-cell responses. Immunology 129: 496–505. doi: 10.1111/j.1365-2567.2009.03201.x PMID: 20002212
13. Axelsson-Robertson R, Loxton AG, Walzl G, Ehlers MM, Kock MM, et al. (2013) A broad profile of co-dominant epitopes shapes the peripheral Mycobacterium tuberculosis specific CD8+ T-cell immune response in South African patients with active tuberculosis. PLoS One 8: e58309. doi: 10.1371/journal.pone.0058309 PMID: 23555576
14. Divangahi M, Desjardins D, Nunes-Alves C, Remold HG, Behar SM (2010) Eicosanoid pathways regulate adaptive immunity to Mycobacterium tuberculosis. Nat Immunol 11: 751–758. doi: 10.1038/ni.1904 PMID: 20622882
15. Nobrega C, Nunes-Alves C, Cerqueira-Rodrigues B, Roque S, Barreira-Silva P, et al. (2013) T cells home to the thymus and control infection. J Immunol 190: 1646–1658. doi: 10.4049/jimmunol.1202412 PMID: 23315077
16. Billeskov R, Vingsbo-Lundberg C, Andersen P, Dietrich J (2007) Induction of CD8 T cells against a novel epitope in TB10.4: correlation with mycobacterial virulence and the presence of a functional region of difference-1. J Immunol 179: 3973–3981. PMID: 17785835
17. Tully G, Kortsik C, Höhn H, Zehbe I, Hitzler WE, et al. (2005) Highly focused T cell responses in latent human pulmonary Mycobacterium tuberculosis infection. J Immunol 174: 2174–2184. PMID: 15699149
18. Du G, Chen CY, Shen Y, Qiu L, Huang D, et al. (2010) TCR repertoire, clonal dominance, and pulmonary trafficking of mycobacterium-specific CD4+ and CD8+ T effector cells in immunity against tuberculosis. The Journal of Immunology 185: 3940–3947. doi: 10.4049/jimmunol.1001222 PMID: 20805423
19. Luo W, Su J, Zhang X-B, Yang Z, Zhou M-Q, et al. (2012) Limited T cell receptor repertoire diversity in tuberculosis patients correlates with clinical severity. PLoS ONE 7: e48117. doi: 10.1371/journal.pone.0048117 PMID: 23110186
20. Jacobsen M, Detjen AK, Mueller H, Gutschmidt A, Leitner S, et al. (2007) Clonal expansion of CD8+ effector T cells in childhood tuberculosis. J Immunol 179: 1331–1339. PMID: 17617626
21. Woodworth JS, Wu Y, Behar SM (2008) Mycobacterium tuberculosis-specific CD8+ T cells require perforin to kill target cells and provide protection in vivo. JImmunol 181: 8595–8603. PMID: 19050279

22. Moon JJ, Chu HH, Pepper M, McSorley SJ, Jameson SC, et al. (2007) Naive CD4(+) T cell frequency varies for different epitopes and predicts repertoire diversity and response magnitude. Immunity 27: 203–213. PMID: 17707129

23. Li H, Ye C, Ji G, Han J (2012) Determinants of public T cell responses. Cell research 22: 33–42. doi: 10.1038/cr.2012.1 PMID: 22212481

24. Correia-Neves M, Waltzinger C, Mathis D, Benoist C (2001) The shaping of the T cell repertoire. Immunity 14: 21–32. PMID: 11163227

25. Chackerian AA, Alt JM, Perera TV, Dascher CC, Behar SM (2002) Dissemination of Mycobacterium tuberculosis is influenced by host factors and precedes the initiation of T-cell immunity. Infection and Immunity 70: 4501–4509. PMID: 12117962

26. Wolf AJ, Desvignes L, Linas B, Banaiee N, Tamura T, et al. (2008) Initiation of the adaptive immune response to Mycobacterium tuberculosis depends on antigen production in the local lymph node, not the lungs. J ExpMed 205: 105–115. PMID: 18158321

27. Gallegos AM, Pamer EG, Glickman MS (2008) Delayed protection by ESAT-6-specific effector CD4+ T cells after airborne M. tuberculosis infection. J Exp Med 205: 2359–2368. doi: 10.1084/jem.20080353 PMID: 18779346

28. Reiley WW, Calayag MD, Wittmer ST, Huntington JL, Pearl JE, et al. (2008) ESAT-6-specific CD4 T cell responses to aerosol Mycobacterium tuberculosis infection are initiated in the mediastinal lymph nodes. ProcNatlAcadSciUSA 105: 10961–10966. doi: 10.1073/pnas.0801496105 PMID: 18667699

29. Ji H, Liu Y, Zhang Y, Shen XD, Gao F, et al. (2014) T-cell immunoglobulin and mucin domain 4 (TIM-4) signaling in innate immune-mediated liver ischemia-reperfusion injury. Hepatology.

30. Lee J, Su EW, Zhu C, Hainline S, Phuah J, et al. (2011) Phosphotyrosine-dependent coupling of Tim-3 to T-cell receptor signaling pathways. Mol Cell Biol 31: 3963–3974. doi: 10.1128/MCB.05297-11 PMID: 21807895

31. Li H, Wu K, Tao K, Chen L, Zheng Q, et al. (2012) Tim-3/galectin-9 signaling pathway mediates T-cell dysfunction and predicts poor prognosis in patients with hepatitis B virus-associated hepatocellular carcinoma. Hepatology 56: 1342–1351. doi: 10.1002/hep.25777 PMID: 22505239

32. Andrews JR, Noubary F, Walensky RP, Cerda R, Losina E, et al. (2012) Risk of progression to active tuberculosis following reinfection with Mycobacterium tuberculosis. Clin Infect Dis 54: 784–791. doi: 10.1093/cid/cir951 PMID: 22267721

33. Qi Q, Liu Y, Cheng Y, Glanville J, Zhang D, et al. (2014) Diversity and clonal selection in the human T-cell repertoire. Proceedings of the National Academy of Sciences 111: 13139–13144. doi: 10.1073/pnas.1409155111 PMID: 25157137

34. Brodin P, Rosenkrands I, Andersen P, Cole ST, Brosch R (2004) ESAT-6 proteins: protective antigens and virulence factors? Trends Microbiol 12: 500–508. PMID: 15488391

35. Majlessi L, Brodin P, Brosch R, Rojas MJ, Khun H, et al. (2005) Influence of ESAT-6 secretion system 1 (RD1) of Mycobacterium tuberculosis on the interaction between mycobacteria and the host immune system. The Journal of Immunology 174: 3570–3579. PMID: 15749894

36. Woodworth JS, Fortune SM, Behar SM (2008) Bacterial protein secretion is required for priming of CD8+ T cells specific for the Mycobacterium tuberculosis antigen CFP10. Infect Immun 76: 4199–4205. doi: 10.1128/IAI.00307-08 PMID: 18591224

37. Baena A, Porcelli S (2009) Evasion and subversion of antigen presentation by Mycobacterium tuberculosis. Tissue Antigens 74: 189–204. doi: 10.1111/j.1399-0039.2009.01301.x PMID: 19563525

38. Comas I, Chakravartti J, Small PM, Galagan J, Niemann S, et al. (2010) Human T cell epitopes of Mycobacterium tuberculosis are evolutionarily hyperconserved. NatGenet 42: 498–503. doi: 10.1038/ng.590 PMID: 20495566

39. Lindenstrom T, Aagaard C, Christensen D, Agger EM, Andersen P (2014) High-frequency vaccine-induced CD8(+) T cells specific for an epitope naturally processed during infection with Mycobacterium tuberculosis do not confer protection. Eur J Immunol 44: 1699–1709. doi: 10.1002/eji.201344358 PMID: 24677089

40. Aagaard CS, Hoang TTKT, Vingsbo-Lundberg C, Dietrich J, Andersen P (2009) Quality and vaccine efficacy of CD4+ T cell responses directed to dominant and subdominant epitopes in ESAT-6 from Mycobacterium tuberculosis. J Immunol 183: 2659–2668. doi: 10.4049/jimmunol.0900947 PMID: 19620314

41. Woodworth JS, Aagaard CS, Hansen PR, Cassidy JP, Agger EM, et al. (2014) Protective CD4 T cells targeting cryptic epitopes of Mycobacterium tuberculosis resist infection-driven terminal differentiation. J Immunol 192: 3247–3258. doi: 10.4049/jimmunol.1300283 PMID: 24574499

42. Yang J, He J, Huang H, Ji Z, Wei L, et al. (2013) Molecular characterization of T cell receptor beta variable in the peripheral blood T cell repertoire in subjects with active tuberculosis or latent tuberculosis infection. BMC Infect Dis 13: 423. doi: 10.1186/1471-2334-13-423 PMID: 24010943

43. Yang J, Xu K, Zheng J, Wei L, Fan J, et al. (2013) Limited T cell receptor beta variable repertoire responses to ESAT-6 and CFP-10 in subjects infected with Mycobacterium tuberculosis. Tuberculosis (Edinb) 93: 529–537. doi: 10.1016/j.tube.2013.05.007 PMID: 23845455

44. Barry CE, Boshoff HI, Dartois V, Dick T, Ehrt S, et al. (2009) The spectrum of latent tuberculosis: rethinking the biology and intervention strategies. Nat Rev Microbiol 7: 845–855. doi: 10.1038/nrmicro2236 PMID: 19855401

45. Lin PL, Rodgers M, Smith L, Bigbee M, Myers A, et al. (2009) Quantitative comparison of active and latent tuberculosis in the cynomolgus macaque model. Infect Immun 77: 4631–4642. doi: 10.1128/IAI.00592-09 PMID: 19620341

46. Nunes-Alves C, Booty MG, Carpenter SM, Jayaraman P, Rothchild AC, et al. (2014) In search of a new paradigm for protective immunity to TB. Nat Rev Microbiol 12: 289–299. doi: 10.1038/nrmicro3230 PMID: 24590243

47. Gorman JV, Starbeck-Miller G, Pham NL, Traver GL, Rothman PB, et al. (2014) Tim-3 directly enhances CD8 T cell responses to acute Listeria monocytogenes infection. J Immunol 192: 3133–3142. doi: 10.4049/jimmunol.1302290 PMID: 24567532

48. Lewinsohn DA, Winata E, Swarbrick GM, Tanner KE, Cook MS, et al. (2007) Immunodominant Tuberculosis CD8 Antigens Preferentially Restricted by HLA-B. PLoS Pathogens 3: e127–1249. PMID: 17677004

49. Lewinsohn DM, Swarbrick GM, Cansler ME, Null MD, Rajaraman V, et al. (2013) Human Mycobacterium tuberculosis CD8 T Cell Antigens/Epitopes Identified by a Proteomic Peptide Library. PLoS ONE 8: e67016. PMID: 23805289

50. Lindestam Arlehamn CS, Gerasimova A, Mele F, Henderson R, Swann J, et al. (2013) Memory T Cells in Latent Mycobacterium tuberculosis Infection Are Directed against Three Antigenic Islands and Largely Contained in a CXCR3+CCR6+ Th1 Subset. PLoS Pathogens 9: e1003130. doi: 10.1371/journal.ppat.1003130 PMID: 23358848

51. Lepore M, Kalinicenko A, Colone A, Paleja B, Singhal A, et al. (2014) Parallel T-cell cloning and deep sequencing of human MAIT cells reveal stable oligoclonal TCRbeta repertoire. Nat Commun 5: 3866. doi: 10.1038/ncomms4866 PMID: 24832684

52. Stewart JJ, Lee CY, Ibrahim S, Watts P, Shlomchik M, et al. (1997) A Shannon entropy analysis of immunoglobulin and T cell receptor. Mol Immunol 34: 1067–1082. PMID: 9519765

53. Sherwood AM, Emerson RO, Scherer D, Habermann N, Buck K, et al. (2013) Tumor-infiltrating lymphocytes in colorectal tumors display a diversity of T cell receptor sequences that differ from the T cells in adjacent mucosal tissue. Cancer Immunol Immunother 62: 1453–1461. doi: 10.1007/s00262-013-1446-2 PMID: 23771160

54. Moon JJ, Chu HH, Hataye J, Pagán AJ, Pepper M, et al. (2009) Tracking epitope-specific T cells. Nat Protoc 4: 565–581. doi: 10.1038/nprot.2009.9 PMID: 19373228

55. Holst J, Vignali KM, Burton AR, Vignali DAA (2006) Rapid analysis of T-cell selection in vivo using T cell-receptor retrogenic mice. Nat Methods 3: 191–197. PMID: 16489336

56. Holst J, Szymczak-Workman AL, Vignali KM, Burton AR, Workman CJ, et al. (2006) Generation of T-cell receptor retrogenic mice. Nat Protoc 1: 406–417. PMID: 17406263
